# Pulmonary artery catheters or central venous catheters for cardiac surgery: the PUMA Pilot randomised clinical trial

**DOI:** 10.1111/anae.70282

**Published:** 2026-06-28

**Authors:** Luke A. Perry, Reny Segal, Marco Larobina, Rinaldo Bellomo, Julian A. Smith, Jesselyn Sin, Katrina Danial, Alistair R. D. McLean, Lisa Q. Rong, Mario Gaudino, Thomas Schwann, Marie Palumbo, Luke O'Halloran, Brian Chee, Jinesh Patel, Andrew Silvers, Jayme Bennetts, Silvana Marasco, Tim G. Coulson, Alistair Royse, Emily See, Lachlan F. Miles, the ANZCA Clinical Trials Network

**Affiliations:** ^1^ Victorian Cardiac Anaesthesia Research Laboratory, Department of Surgery, School of Clinical Sciences Monash University Melbourne VIC Australia; ^2^ Department of Anaesthesia and Perioperative Medicine Victorian Heart Hospital Melbourne VIC Australia; ^3^ Department of Critical Care, Melbourne Medical School The University of Melbourne Melbourne VIC Australia; ^4^ Department of Anaesthesia and Pain Management Royal Melbourne Hospital Melbourne VIC Australia; ^5^ Department of Cardiothoracic Surgery Victorian Heart Hospital, Monash Health Melbourne VIC Australia; ^6^ Department of Surgery, School of Clinical Sciences at Monash Health Monash University Melbourne VIC Australia; ^7^ Centre for Epidemiology and Biostatistics, Melbourne School of Population and Global Health The University of Melbourne Melbourne VIC Australia; ^8^ MISCH (Methods and Implementation Support for Clinical Health) Research Hub, Faculty of Medicine, Dentistry and Health Sciences The University of Melbourne Melbourne VIC Australia; ^9^ Department of Anesthesiology Weill Cornell Medicine New York NY USA; ^10^ Department of Cardiothoracic Surgery Weill Cornell Medicine New York NY USA; ^11^ Department of Cardiovascular Surgery Corewell Health Royal Oak MI USA; ^12^ Cardiothoracic Unit The Alfred Melbourne VIC Australia; ^13^ Department of Anaesthesiology and Perioperative Medicine Alfred Health Melbourne VIC Australia; ^14^ Department of Cardiothoracic Surgery Royal Melbourne Hospital Melbourne VIC Australia; ^15^ Department of Surgery The University of Melbourne Melbourne VIC Australia; ^16^ Departments of Intensive Care and Nephrology The Royal Melbourne Hospital Melbourne VIC Australia; ^17^ Department of Anaesthesia Austin Health Melbourne VIC Australia

**Keywords:** cardiac anaesthesia, cardiac surgery, feasibility studies, haemodynamic monitoring, pulmonary artery catheter

## Abstract

**Introduction:**

Pulmonary artery catheters are used widely in cardiac surgery despite observed associations with worse outcomes and guidelines that recommend against their routine use. No adequately powered randomised trials are available.

**Methods:**

The PUMA Pilot was a multicentre, randomised, parallel assignment, open‐label, pilot and feasibility trial conducted at three tertiary cardiac surgery centres. Eligible patients were adults undergoing coronary artery bypass grafting, aortic valve replacement or surgery on the aortic root or ascending aorta with or without aortic valve replacement, with a predicted surgical mortality of < 2%. Patients were allocated randomly to receive a pulmonary artery catheter or a central venous catheter inserted immediately before surgery. The primary feasibility outcome was protocol compliance, defined as receiving the assigned intervention without crossover. Secondary feasibility outcomes were eligibility rate; recruitment proportion and rate; data completeness; and rate of clinician refusal.

**Results:**

We screened 480 patients and 206 (43%) were eligible; 150/203 (74%) approached provided informed consent. Three of 206 (1%) eligible patients were not included due to clinician refusal. Of 149 patients who were randomised, 76 were assigned to the pulmonary artery catheter group and 73 to the central venous catheter group. For the primary feasibility outcome, 147 patients (99%) received the allocated intervention. Data were complete for 144 (97%) patients. Median (IQR [range]) days alive and at home at 30 days was 23.7 (21.9–24.7 [7.0–26.0]) in the pulmonary artery catheter group and 22.9 (20.8–23.9 [8.6–25.8]) in the central venous catheter group. Acute kidney injury occurred in 26/76 (34%) patients in the pulmonary artery catheter group and 14/73 (19%) in the central venous catheter group.

**Discussion:**

A randomised trial of pulmonary artery catheters compared with central venous catheters in low‐risk cardiac surgery is feasible. Such a trial would address significant practice variability and inform international guidelines.

## Plain Language Summary

Researchers carried out a pilot study in three hospitals to see whether it would be possible to run a much larger trial. They compared two different types of devices used to monitor patients during heart surgery: a pulmonary artery catheter and a central venous catheter. Almost 150 patients having low‐risk heart surgery were randomly assigned to receive one of the two monitoring methods.

**Why did we do it:**

Doctors around the world use pulmonary artery catheters during heart surgery, but there is debate about whether they help patients. Some studies have even suggested they might be linked to worse outcomes. Because there have not been any large, high‐quality trials to answer this question, the researchers first needed to find out whether a bigger study could be carried out successfully.

**What we found:**

The study showed that a larger trial would be possible. Most eligible patients agreed to take part, very few doctors refused participation and almost every patient received the monitoring method they had been assigned. The researchers were able to collect nearly all of the information they needed. Patients in both groups spent a similar amount of time alive and at home after surgery. However, kidney problems occurred more often in patients who received a pulmonary artery catheter. The results suggest that a larger study is needed to find out whether one monitoring method is safer or better than the other.

## Introduction

Large, randomised trials have evaluated pulmonary artery catheters across a range of critical care settings including sepsis; acute respiratory distress syndrome; general intensive care; and major noncardiac surgery – no benefits to patients have been reported [1, 2, 3, 4]. Annual pulmonary artery catheter use in the USA peaked in 2001 at 1.2 million devices inserted with a total cost exceeding £1.48 billion ($1.98 billion, €1.71 billion) [5]. Following calls for a moratorium [6], their use in several clinical scenarios decreased by approximately 90% over the subsequent 15 years [7, 8]. Cardiac surgery remains an outlier, both for ongoing widespread use of pulmonary artery catheters and for a lack of data from high‐quality randomised trials [9].

The role of pulmonary artery catheters in contemporary cardiac surgery remains controversial due to uncertain benefit and the possibility of rare but catastrophic complications [10, 11, 12]. While they generate useful insights into cardiovascular physiology – which can be used to guide treatment – they may also be associated with unintended harm caused by over‐ or misdiagnosis of abnormal physiology; triggering unnecessary interventions; and delaying discharge from the ICU [9, 13]. Moreover, the accuracy and precision of the thermodilution cardiac index have been challenged, raising concerns about clinical decision‐making based on potentially flawed haemodynamic data [12, 14, 15]. Central venous catheters, which follow the same insertion path but terminate approximately 25 cm shallower at the cavo‐atrial junction, hold potential safety and economic advantages over pulmonary artery catheters, particularly in low‐risk populations where direct pulmonary arterial pressure monitoring is not required.

Observational studies have reported conflicting associations between pulmonary artery catheter use and patient outcomes. A recent systematic review of seven non‐randomised studies reported an increased risk of operative mortality and other complications when pulmonary artery catheters were used [9], while a subsequent large observational study reported a survival benefit [16]. Clinical equipoise is reflected in the variable uptake of best practice guidelines that recommend against routine use in low‐risk cases [17, 18, 19] and international surveys and epidemiologic studies have reported extensive practice heterogeneity based on geographical and institutional, rather than patient‐level, factors [20, 21, 22, 23]. Persistent use in some regions may reflect the low‐quality, observational and conflicting nature of the available evidence; the perceived value of pulmonary artery catheters for training and maintenance of competence among cardiac anaesthetists; and the possibility that institutional experience with pulmonary artery catheter use may modify their risks and benefits.

A definitive randomised trial is required to address ongoing uncertainty. The PUMA trial was designed to confirm whether such a trial is feasible and to generate data to inform its design.

## Methods

The PUMA trial was a multicentre, pragmatic, randomised, pilot and feasibility trial comparing peri‐operative management with a pulmonary artery catheter to a central venous catheter in adults undergoing low‐risk cardiac surgery conducted at three Australian sites. The trial was endorsed by the Australian and New Zealand College of Anaesthetists Clinical Trials Network. All sites routinely used pulmonary artery catheters in > 80% of cases in the 12 months before the trial. Ethics approval was granted by the Royal Melbourne Hospital Human Research Ethics Committee. Written informed consent was obtained from all patients. This trial was co‐designed with patients. This trial was conducted and is reported according to CONSORT statement extension for pilot and feasibility trials [24, 25]. The trial protocol is available in online Supporting Information Appendix S1.

Adults (aged ≥ 18 y) undergoing coronary artery bypass grafting, isolated surgical aortic valve replacement or repair, or surgery on the aortic root or ascending aorta with or without aortic valve replacement were included in this trial. Exclusion criteria were: European System for Cardiac Operative Risk Evaluation (EuroSCORE 2) predicted operative mortality > 2% [26]; emergency surgery (defined as a decision‐to‐operation time of < 1 business day); repeat sternotomy; pulmonary hypertension defined on pre‐operative echocardiography or right heart study as right ventricular systolic pressure > 35 mmHg or a mean pulmonary artery pressure > 25 mmHg; left ventricular ejection fraction < 30%; any degree of pre‐operative right ventricular systolic impairment; and right heart structural abnormality that was a contraindication to using a pulmonary artery catheter.

After eligibility and consent were confirmed, patients were allocated randomly (1:1) to receive a pulmonary artery or central venous catheter. An independent statistician generated the randomisation sequence using randomly permuted blocks stratified by trial site and surgical urgency. This was uploaded to a secure, central platform. Investigators and clinical staff had no access to the randomisation module and treatment allocation was only released after the enrolment procedure was complete. To avoid bias due to a case being cancelled, patients were randomised < 24 h before the anticipated time of surgery. Blinding of patients and treating clinicians was not feasible; however, outcome assessors were blinded to the treatment allocation.

Patients had either a pulmonary artery catheter or a central venous catheter inserted before the start of surgery in accordance with local policies and procedures. Goal‐directed treatment algorithms were not part of the study protocol, to reflect diverse real‐world practice and maximise external generalisability. The manufacturer, model, size, insertion site and how data was obtained and used from the interventions were at the discretion of treating clinicians. Patients allocated to receive a central venous catheter could receive a pulmonary artery catheter at any point if the treating clinicians determined one was required. Use of other advanced haemodynamic monitoring devices was not restricted in either group.

The primary feasibility outcome was treatment crossover, as emergency crossover to the pulmonary artery catheter group in patients who were deteriorating was considered a principal threat to feasibility. Secondary feasibility outcomes included eligibility rate; recruitment proportion; recruitment rate; complete case report form rate; and the proportion of eligible patients who were not enrolled due clinician refusal. Minimum targets for each feasibility outcome were set a priori (online Supporting Information Table S1).

The primary pilot clinical outcome was days alive and at home at 30 days (DAH_30_). Key secondary pilot clinical outcomes were: acute kidney injury (AKI) according to Kidney Disease Improving Global Outcomes criteria occurring during the postoperative admission [27]; all‐cause mortality at 30 and 90 days; change in European 5‐level, 5‐dimension quality of life score (EQ‐5D‐5L) [28] from baseline to 90 days; time in postoperative organ dysfunction at 30 days (TPOD_30_; defined as the cumulative duration where ≥ 1 of the following treatments were active: vasoactive infusions, mechanical circulatory support, mechanical ventilation, or renal replacement therapy, measured in hours); and duration of postoperative admissions in the ICU and hospital censored at postoperative day 30. Adverse events related to device insertion and use were assessed. A complete list of the exploratory clinical and safety outcomes is in online Supporting Information Appendix S2.

Data on baseline characteristics, surgical procedure, peri‐operative care and feasibility and pilot clinical outcomes were collected. A trained assessor masked to treatment allocation performed telephone follow up at 30 and 90 days after surgery.

PUMA was designed to evaluate the feasibility of conducting a definitive trial rather than test clinical hypotheses. For each feasibility outcome, we specified a prior estimate of the expected proportion and the minimum feasibility threshold necessary to proceed to a definitive trial (online Supporting Information Table S1). Based on the primary feasibility outcome, a sample size of 137 patients was calculated to provide 90% power that the lower bound of the binomial exact (Clopper‐Pearson) 95%CI for the observed proportion would exceed the corresponding feasibility threshold, assuming the true proportion equalled its previous estimate. To account for drop out or clinician refusal, the sample size was increased to 150 patients.

Feasibility outcomes were presented as proportions with 95% binomial exact (Clopper‐Pearson) CIs. Binary clinical outcomes were analysed using generalised linear models with a Poisson distribution with a log link and robust standard errors to estimate risk ratios (RR) and 95%CI. Stratification factors (trial site and surgical urgency) were included as model covariates. Two‐sided p values were obtained from Wald tests. Continuous secondary outcomes were compared using quantile regression (τ = 0.5) and presented as median differences with 95%CIs.

Due to the exploratory nature of analyses of clinical outcomes, no adjustments were made for multiple comparisons. All analyses on these outcomes were conducted on an intention‐to‐treat basis, evaluating treatment policy estimates. Across all clinical outcomes, we conducted complete case analyses whereby patients with missing data for a specific outcome were excluded from that specific analysis. No imputation was performed for missing data. All analyses were conducted using R version 4.4.3 (R Foundation for Statistical Computing, Vienna, Austria).

## Results

**Figure 1:**
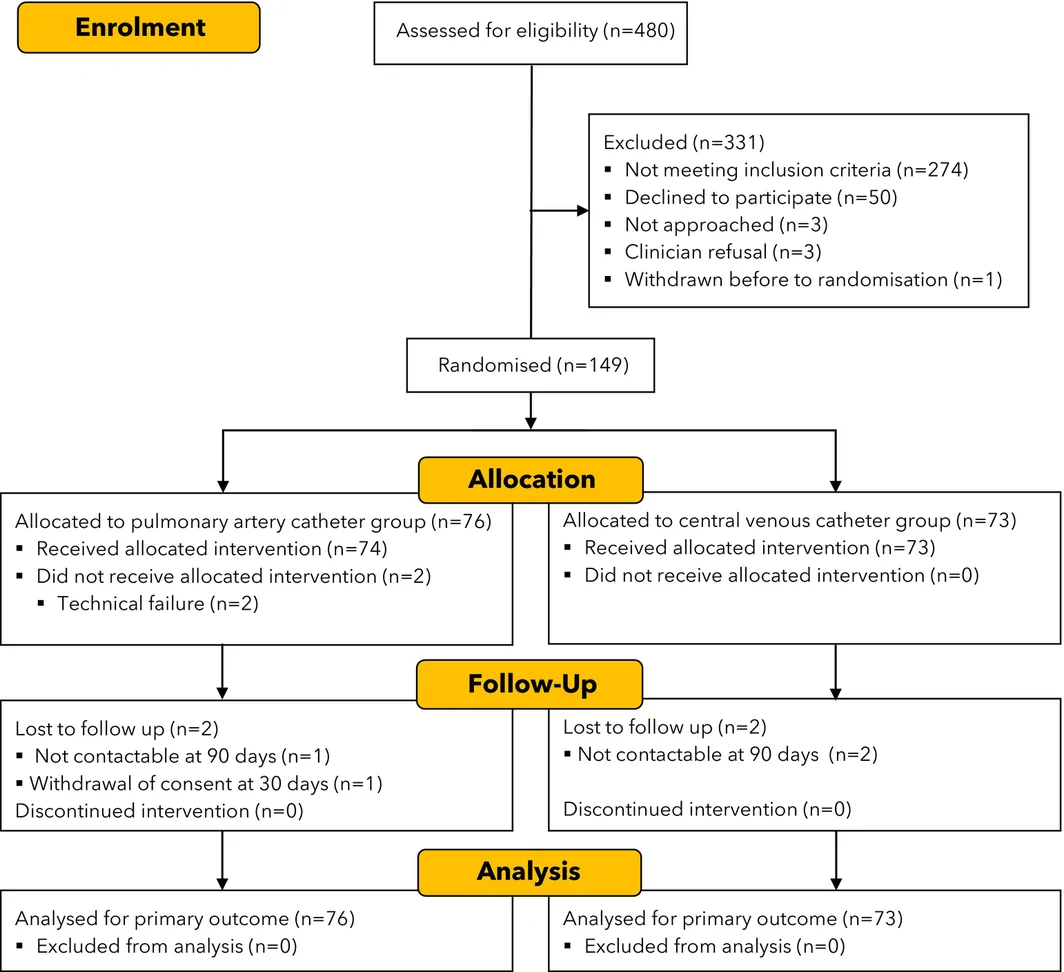
Study flow diagram.

From July 2024 to February 2025, 480 patients were screened, of whom 206 were eligible to participate (Fig. 1). Participation was proposed to 203 patients, of whom 150 provided informed consent and were enrolled into the trial. One enrolled patient was not randomised inadvertently before surgery due to a database coding error and was withdrawn from the trial. Of the remaining patients, 76 were assigned randomly to the pulmonary artery catheter group and 73 to the central venous catheter group. Baseline demographic and clinical characteristics were similar between the two groups (Table 1 and online Supporting Information Table S2).

**Table 1:** Baseline patient characteristics. Values are median (IQR [range]) or number (proportion).

	Pulmonary artery catheter group	Central venous catheter group
	n = 76	n = 73
Age; y	66 (57–70 [24–80])	64 (58–72 [35–83])
Sex; female	8 (11%)	10 (14%)
BMI; kg.m^−2^	28 (26–32 [19–44])	28 (25–32 [20–61])
Surgery type		
Cardiac artery bypass graft	70 (92%)	68 (93%)
Aortic valve replacement	6 (8%)	5 (7%)
Urgency		
Elective	39 (51%)	40 (55%)
Urgent	37 (49%)	33 (45%)
EuroSCORE 2	0.94 (0.69–1.26 [0.50–1.98])	0.97 (0.79–1.29 [0.50–1.89])
Cardiopulmonary bypass	73 (96%)	70 (96%)
Pre‐operative left ventricular ejection fraction		
> 50%	68 (90%)	63 (86%)
31–50%	8 (11%)	10 (14%)

There was no treatment crossover from the central venous catheter group to the pulmonary artery catheter group. Two patients (3%) allocate to the pulmonary artery catheter group did not receive their assigned intervention due to technical difficulties encountered during insertion and were instead managed with central venous catheters alone (Table 2).

**Table 2:** Feasibility outcomes. Values are number, mean or proportion.

Feasibility outcome	Previous estimate	Feasibility threshold	Observed estimate (95%CI)
Protocol compliance	90%	80%	147/149; 98.7% (96.3–99.8)
Eligibility rate	40%	30%	206/480; 42.9% (38.4–47.5)
Recruitment proportion	60%	30%	150/206; 72.8% (66.2–78.8)
Recruitment rate; patients/site/month	12	8	9.7 (9.3–11.4)
Complete case report form proportion	99%	95%	145/149; 96.7% (92.4–98.9)
Acceptability	85%	70%	98.0% (94.4–99.6)

The eligibility rate, recruitment proportion, recruitment rate, complete case report form rate and clinician refusal exceeded prespecified feasibility thresholds (Table 2). Three of 206 eligible patients (2%) were subsequently not included due to clinician refusal: one due to surgical complexity; one due to a left‐sided superior vena cava; and one in whom transoesophageal echocardiography was contraindicated. Case report form completion was satisfactory, with 3/149 (2%) patients lost to follow up at 90 days, and 1/149 patient who withdrew consent before the 30 day follow up.

Acute kidney injury occurred in 26/76 (34%) patients allocated to the pulmonary artery catheter group and 14/73 (19%) patients allocated to the central venous catheter group (RR 1.80, 95%CI 1.03–3.14). No significant differences were observed for any other key pilot clinical outcome (Table 3). Results for all pilot clinical outcomes are reported in online Supporting Information Tables S3–S9.

**Table 3:** Key pilot clinical outcomes. Values are number (proportion) or median (IQR [range]).

	Pulmonary artery catheter group	Central venous catheter group	Risk ratio (95%CI)	p value
	n = 76	n = 73		
Acute kidney injury	26 (34%)	14 (19%)	1.80 (1.03–3.14)	0.04
All‐cause mortality at 90 days	0/75	0/71	‐	‐

			**Median difference (95%CI)**	
DAH_30_; days	23.7 (21.9–24.7 [7.0–26.0])	22.9 (20.8–23.9 [8.7–25.8])	0.78 (‐0.44–2.01)	0.21
TPOD_30_; h	16.9 (9.6–26.0 [2.5–160.0])	15.6 (9.4–26.7 [2.4–200.0])	1.27 (‐2.50–5.04)	0.51
Duration of ICU stay; h	25.0 (22.0–30.0 [13.0–236.0])	28.0 (22.0–50.0 [16.0–189.00])	‐3.00 (‐9.83–3.83)	0.39
Duration of hospital stay; days	6.3 (5.3–7.3 [4.0**–**17.3])	6.7 (6.0–8.1 [3.9–21.3])	‐3.00 (‐26.58–20.58)	0.80
Change in EQ‐5D‐5L VAS at 90 days	10.0 (0–25.0 [‐25.0–50.0])	8.0 (0–20.0 [‐30.0–58.0])	2.00 (‐3.98–7.98)	0.51

DAH30, days alive at home 30 days; TPOD_30_, time in peri‐operative organ dysfunction at 30 days; EQ‐5D‐5L VAS, European 5‐level, 5‐dimension visual analogue scale (0–100).

An inadvertent arterial puncture occurred in one patient allocated to the pulmonary artery catheter group without further complication, and one patient allocated to the central venous catheter group represented after discharge with a subacute nonocclusive thrombus in the right internal jugular vein that did not require treatment. No other adverse device‐related events were reported.

## Discussion

The PUMA randomised pilot clinical trial has shown that, in adults undergoing low‐risk cardiac surgery, a large, multicentre clinical trial comparing pulmonary artery catheters with central venous catheters is feasible. Within the limits of the sample size, the absence of emergency treatment crossover and serious adverse device events confirms it would be safe to conduct such a trial. Excellent trial fidelity and minimal barriers to individual patient participation determine the clinical acceptability of both interventions at sites that insert pulmonary artery catheters in a high proportion of patients.

As a small pilot trial, PUMA was not powered to detect differences in clinical outcomes. No between‐group differences in exploratory clinical outcomes including DAH_30_, time in postoperative organ dysfunction at 30 days (TPOD_30_), all‐cause mortality at 90 days and change in EQ‐5D‐5L from baseline to 90 days were observed. Acute kidney injury occurred more frequently in patients allocated to the pulmonary artery catheter group. Such an association was observed previously by an observational study [29], and there is a plausible mechanistic link to the interventions through their potential influence on peri‐operative haemodynamic management. However, this result should be interpreted with caution and be considered hypothesis‐generating only, given the small sample size, potential for chance imbalances in prognostic factors, lack of adjustment for multiplicity in the context of several exploratory outcomes and its statistical fragility (fragility index = 1). Nevertheless, it appears reasonable for AKI to be included as an outcome in subsequent randomised trials.

A recent systematic review confirmed the lack of high‐quality randomised trials in cardiac surgery [9]. A small, single‐centre trial (n = 60) compared pulmonary artery catheters to central venous catheters in off‐pump coronary artery bypass surgery and found no difference in outcomes [30]. Two other small, single‐centre trials (n = 58 and n = 40) compared goal‐directed management with pulmonary artery catheters to active controls (transpulmonary thermodilution cardiac output monitor and a novel bioreactance monitor) and reported an increase in duration of mechanical ventilation in patients in the pulmonary artery catheter group [31, 32]. Conversely, major noncardiac surgery has seen several large, randomised trials of pulmonary artery catheters which have failed to show patient benefit [1, 3]. The absence of a definitive trial in patients undergoing cardiac surgery, despite calls for such evidence [18], highlights the importance of this pilot and feasibility trial as an essential prerequisite to a landmark trial [33].

The PUMA trial establishes the feasibility of a larger trial across a range of important feasibility outcomes. It also suggests that such a trial is safe; there were no emergency crossover events to the pulmonary artery catheter group and no serious adverse device‐related adverse events. These results can be used to inform the design and sample size calculation of future trials. Such trials must consider several design challenges. First, choosing eligibility criteria that optimally balance safety and acceptability against external generalisability. While the present trial used a conservative predicted operative mortality cutoff of 2%, other trials have defined the cutoff for low‐risk at < 4%, most notably the PARTNER‐3 trial [34]. Second, an international trial should consider the generalisability of different risk models including the EuroSCORE and Society of Thoracic Surgeons Operative Risk Calculator [35]. Third, whether to use a non‐inferiority or superiority design; central venous catheters hold potential safety advantages over pulmonary artery catheters including lower rates of rare but catastrophic complications, shorter stays in the ICU and lower costs. Accordingly, if central venous catheters are shown to be non‐inferior in a large trial, these advantages and historic responses to neutral pulmonary artery catheter trials would be adequate grounds for de‐adoption. Finally, ensuring appropriate strategies are in place to ensure equitable representation of women, including expanding eligibility to include more valve surgeries, in which women are better represented [36], and consideration of novel methods such as targeted extension phases pioneered by the ROMA Women trial [37].

This pilot trial has several strengths. First, the choice of a pragmatic design over a goal‐directed treatment algorithm reflects the real‐world practice of experienced clinicians, improves external generalisability and enables nuanced clinical decision‐making based on diverse sources of information. Second, including hospitals with significant experience with pulmonary artery catheters establishes the feasibility of a large trial in a broad range of settings, including in the United States, where the median utilisation rate of pulmonary artery catheters is 80% [38]. Third, it was powered appropriately based on a primary feasibility outcome, which is best practice for pilot and feasibility trials [25].

This trial also has several limitations. First, there is no universally accepted definition of ‘low‐risk’ in cardiac surgery, and the eligibility criteria were optimised for acceptability and feasibility in sites with high pulmonary artery catheter use, perhaps at the cost of external generalisability. Second, while the recruitment rate was high (approximately 10 patients/site/month on average), this might not be a realistic for sites in countries with a variable case load and mix. Third, most included cases were coronary artery bypass surgeries, and the feasibility of enrolling aortic valve and proximal aortic cases is less clear, given their lower frequency and the speed of trial recruitment. Importantly, patients undergoing mitral valve surgery were excluded from this trial. Subsequent trials must consider the potential risks and benefits of expanding surgical case‐mix eligibility, ideally in partnership with surgical, anaesthesia and critical care focus groups. Fourth, and consistent with other studies in cardiac surgery [39], women were underrepresented, comprising only 12% of the study population. Fifth, a database coding error led to a single enrolled patient not being randomised before surgery; however, this did not lower the sample size below that which was defined as adequate a priori (n = 137). Finally, all sites had considerable experience with pulmonary artery catheters before the start of the trial. Accordingly, while this trial showed that managing low‐risk patients without a pulmonary artery catheter was acceptable in high‐use sites, it could not explore acceptability for low‐use sites to manage such patients with a pulmonary artery catheter.

In this multicentre pilot randomised trial comparing pulmonary artery with central venous catheters in adults undergoing low‐risk cardiac surgery, the feasibility of a definitive trial was shown. A large, multicentre, international trial is now required to evaluate the safety and effectiveness of pulmonary artery catheters in cardiac surgery.

## Supporting information


**Appendix S1.** The PUMA pilot protocol.

**Appendix S2.** List of pilot clinical and safety outcomes.

**Table S1.** Expected precision for feasibility outcomes.
**Table S2.** Baseline patient characteristics.
**Table S3.** Procedural events.
**Table S4.** Major complications.
**Table S5.** Allogeneic blood products and albumin.
**Table S6.** Diuretics, fluid balance and lactate.
**Table S7.** Vasopressor and inotrope requirements.
**Table S8.** Course of recovery.
**Table S9.** Patient‐centred outcomes.
