# Supplementary material for: Pulmonary artery catheters or central venous catheters for cardiac surgery: the PUMA Pilot randomised clinical trial
Source: Anaesthesia. 2026 Jun 28;81(10):1358–66. doi: 10.1111/anae.70282 (PMC13551237; doi:10.1111/anae.70282)
Supplement: Supplementary file 1 [file ANAE-81-1358-s001.docx]

|  | | | | |
| --- | --- | --- | --- | --- |
|  |  | |  |  |
|  |  | |  |  |
| TRIAL PROTOCOL^[1]^ | |  | | |
| Scientific Title | | Pulmonary artery catheters in adults undergoing low risk cardiac surgery (The PUMA Pilot): a multicentre, randomised, controlled, consumer co-designed, Vanguard, pilot and feasibility clinical trial. | | |
| Sponsor | | Melbourne Health | | |
| Endorsements | | ANZCA Clinical Trial Network | | |
| Sites | | Royal Melbourne Hospital  Melbourne Private Hospital  Victorian Heart Hospital, Monash Health  Jessie McPherson Private Hospital  Alfred Health  Austin Health  Weill Cornell Medical Center  University of Michigan Cardiovascular Center  Corewell Health | | |
| Funders | | Heart Foundation  ANZCA Foundation | | |
| Principal Investigator | | Dr Luke Perry | | |
| ANZCTR Identifier | | ACTRN12623000522617p | | |
| HREC Numbers | | 2023.044 (RMH) | | |
| Version Number and Date | | 2.2, 14 July 2024 | | |

**SUMMARY OF CHANGES IN PROTOCOL 2.1**

Change 1: addition of new sites: (1) Weill-Cornell Medical Center; (2) University of Michigan Cardiovascular Center; (3) Corewell Health; (4) Austin Health; and (5) Alfred Health.

Change 2: the following investigators were added:

**New Chief Investigators**

Prof Mario Gaudino

A/Prof Lisa Rong

Dr Thomas Schwann

Prof Jonathan Haft

Prof Jayme Bennetts

Prof Silvana Marasco

Dr Tim Coulson

A/Prof Andrew Silvers

Change 3: minor changes throughout the protocol to reflect that these new sites and Chief Investigators have been added.

**SUMMARY OF CHANGES IN PROTOCOL 2.0**

Change 1: addition of surgical urgency (i.e. elective or urgent) as a randomisation stratum to ensure interventions are distributed proportionately among elective and urgent cases.

Change 2: addition of new inclusion criteria ‘surgery on the ascending aorta or aortic root with or without aortic valve replacement’ to increase external generalisability and trial feasibility. Note that these patients must still meet strict exclusion criteria and have a Euroscore II < 2% predicted mortality.

Change 3: addition of a new pilot/clinical outcome ‘Sequential Organ Failure Assessment (SOFA) Score on days 1-7’ to enable us to contextualise differences in the intensity of perioperative treatments against the extent of physiological derangement.

Change 4: addition of a new pilot/clinical outcome ‘maximum vasopressor and inotrope dose on postoperative days 1-7’. The cumulative daily dose is already included as an outcome, however because this is time-consuming to calculate, we now include the maximum doses to enable us to compare which outcome is more feasible and acceptable for PUMA II.

**Please note that all changes in Protocol Version 2.0 were enacted before the first patient was recruited to PUMA Pilot.**

These changes were approved by the Royal Melbourne Hospital Human Research Ethics Committee on 21 June 2024.

CONTENTS

[1	INVESTIGATOR LIST	6](#_Toc126745618)

[2	PERSONNEL, COMMITTEES, AND RESPONSIBILITIES	8](#_Toc126745619)

[2.1	Steering Committee	8](#_Toc126745620)

[2.2	Patient Safety Committee	8](#_Toc126745621)

[2.3	Site Chief Investigators	9](#_Toc126745622)

[2.4	Additional Personnel	9](#_Toc126745623)

[3	TRIAL APPROVALS AND REGISTRATION	10](#_Toc126745624)

[3.1	TRIAL NAME	10](#_Toc126745625)

[3.2	SPONSORING INSTITUTION	10](#_Toc126745626)

[3.3	ENDORSEMENTS	10](#_Toc126745627)

[3.4	TRIAL FUNDERS	10](#_Toc126745628)

[3.5	TRIAL REGISTRATION	10](#_Toc126745629)

[3.6	PEER REVIEW	10](#_Toc126745630)

[3.7	HUMAN RESEARCH ETHICS COMMITTEE APPROVALS	11](#_Toc126745631)

[4	LIST OF ABBREVIATIONS	12](#_Toc126745632)

[5	TRIAL SUMMARIES	13](#_Toc126745633)

[5.1	PROFESSIONAL SUMMARY	13](#_Toc126745634)

[5.2	PLAIN LANGUAGE SUMMARY	15](#_Toc126745635)

[6	INTRODUCTION	17](#_Toc126745636)

[6.1	BACKGROUND	17](#_Toc126745637)

[6.1.1	Epidemiology and outcomes in cardiac surgery	17](#_Toc126745638)

[6.1.2	An abbreviated history of the pulmonary artery catheter	17](#_Toc126745639)

[6.1.3	Overview of data obtained from pulmonary artery catheters	18](#_Toc126745640)

[6.1.4	Evidence for pulmonary artery catheter use: non-cardiac surgery	18](#_Toc126745641)

[6.1.5	Evidence for pulmonary artery catheter use: cardiac surgery	19](#_Toc126745642)

[7	OBJECTIVES	24](#_Toc126745643)

[7.1	Aim	24](#_Toc126745644)

[7.2	Hypothesis	24](#_Toc126745645)

[8	TRIAL DESIGN	24](#_Toc126745646)

[8.1	SETTING	24](#_Toc126745647)

[8.2	PILOT NATURE OF THIS STUDY	24](#_Toc126745648)

[8.3	TRIAL TIMELINE	24](#_Toc126745649)

[8.4	TRIAL FLOWCHART	25](#_Toc126745650)

[8.5	DEFINING THE PATIENT JOURNEY	25](#_Toc126745651)

[9	TRIAL POPULATION	26](#_Toc126745652)

[9.1	NUMBER OF PARTICIPANTS	26](#_Toc126745653)

[9.2	INCLUSION CRITERIA	26](#_Toc126745654)

[9.3	EXCLUSION CRITERIA	26](#_Toc126745655)

[9.4	CO-ENROLMENT	27](#_Toc126745656)

[10	PARTICIPANT SELECTION AND ENROLMENT	27](#_Toc126745657)

[10.1	SCREENING	27](#_Toc126745658)

[10.2	INFORMED CONSENT	27](#_Toc126745659)

[10.2.1	Process	27](#_Toc126745660)

[10.2.2	Responsibilities	28](#_Toc126745661)

[10.2.3	Documentation	29](#_Toc126745662)

[11	INTERVENTIONS	29](#_Toc126745663)

[11.1	PULMONARY ARTERY CATHETERS	29](#_Toc126745664)

[11.2	COMPARATOR	29](#_Toc126745665)

[12	OUTCOMES	30](#_Toc126745666)

[12.1	FEASIBILITY OUTCOMES	30](#_Toc126745667)

[12.1.1	Primary feasibility outcome	30](#_Toc126745668)

[12.1.2	Secondary feasibility outcomes	30](#_Toc126745669)

[12.2	PILOT OUTCOMES	31](#_Toc126745670)

[12.2.1	Primary clinical outcome	31](#_Toc126745671)

[12.2.2	Secondary clinical outcomes	31](#_Toc126745672)

[13	ASSIGNMENT OF INTERVENTIONS	34](#_Toc126745673)

[13.1	ALLOCATION SEQUENCE GENERATION	34](#_Toc126745674)

[13.2	ALLOCATION CONCEALMENT	35](#_Toc126745675)

[13.3	IMPLEMENTATION OF TREATMENT ALLOCATION	35](#_Toc126745676)

[13.4	BLINDING	35](#_Toc126745677)

[13.5	CROSSOVER EVENTS	35](#_Toc126745678)

[14	WITHDRAWAL OF PARTICIPANTS	35](#_Toc126745679)

[15	TRIAL PROCEDURES	35](#_Toc126745680)

[15.1	Summary	35](#_Toc126745681)

[15.2	Data Collection and Patient Observations	36](#_Toc126745682)

[15.3	Pre-operative period	36](#_Toc126745683)

[15.4	Day of Surgery	36](#_Toc126745684)

[15.5	Post-operative Day 1	37](#_Toc126745685)

[15.6	Post-operative Day 3	37](#_Toc126745686)

[15.7	ICU Discharge	37](#_Toc126745687)

[15.8	Hospital Discharge	37](#_Toc126745688)

[15.9	Post-operative Day 30	37](#_Toc126745689)

[15.10	Post-operative day 90	37](#_Toc126745690)

[16	DATA MANAGEMENT PLAN	37](#_Toc126745691)

[16.1	Processes	37](#_Toc126745692)

[16.2	Data Variables Collected	38](#_Toc126745693)

[16.2.1	Baseline Data	38](#_Toc126745694)

[16.2.2	Comorbidities	38](#_Toc126745695)

[16.2.3	Regular medications	39](#_Toc126745696)

[16.2.4	Surgical details	39](#_Toc126745697)

[16.2.5	Perioperative data	39](#_Toc126745698)

[16.2.6	Line data	39](#_Toc126745699)

[16.2.7	ICU Data	39](#_Toc126745700)

[16.2.8	Day 30	40](#_Toc126745701)

[16.2.9	Day 90	40](#_Toc126745702)

[16.2.10	Feasibility data	40](#_Toc126745703)

[16.2.11	Safety data	40](#_Toc126745704)

[16.3	Ethical considerations relating to specific types of data to be collected	41](#_Toc126745705)

[16.4	Source Data Identification Log	41](#_Toc126745706)

[16.5	Data Monitoring Plan	41](#_Toc126745707)

[16.6	Data Sharing and Access Plan	42](#_Toc126745708)

[17	SAFETY PROCEDURES	42](#_Toc126745709)

[17.1	Background	42](#_Toc126745710)

[17.2	Definitions	44](#_Toc126745711)

[The following definitions are provided in the National Health and Medical Research Council’s ‘Safety monitoring and reporting in clinical trials involving therapeutic goods’ document and ISO 14155:2020 ‘Clinical investigation of medical devices for human subjects — Good clinical practice’.^62, 63^	44](#_Toc126745712)

[17.3	List of potential adverse events	45](#_Toc126745713)

[17.3.1	Complications associated with central venous puncture (applies to PAC and CVAD)	45](#_Toc126745714)

[17.3.2	Complications associated with PAC use	45](#_Toc126745715)

[17.4	Identifying adverse events	46](#_Toc126745716)

[17.5	Recording and reporting adverse events	46](#_Toc126745717)

[17.6	Assessment of adverse events – Flow Chart	47](#_Toc126745718)

[17.6.1	Assessment of seriousness	47](#_Toc126745719)

[17.6.2	Assessment of causality	47](#_Toc126745720)

[17.6.3	Assessment of expectedness	47](#_Toc126745721)

[17.7	Regulatory reporting requirements	48](#_Toc126745722)

[18	STATISTICS AND DATA ANALYSIS	49](#_Toc126745723)

[18.1	SAMPLE SIZE CALCULATION	49](#_Toc126745724)

[18.2	STATISTICAL ANALYSIS PLAN	49](#_Toc126745725)

[18.3	MISSING DATA	49](#_Toc126745726)

[19	ETHICAL CONDUCT	49](#_Toc126745727)

[19.1	GUIDING PRINCIPLES	49](#_Toc126745728)

[19.2	ETHICAL APPROVALS	50](#_Toc126745729)

[19.3	GOOD CLINICAL PRACTICE TRAINING	50](#_Toc126745730)

[20	CONSUMER INVOLVEMENT	50](#_Toc126745731)

[21	END OF THE TRIAL	51](#_Toc126745732)

[22	ACCESS TO DATA	51](#_Toc126745733)

[23	DISSEMINATION STRATEGY	51](#_Toc126745734)

[23.1	COMMUNICATION OF RESULTS TO PARTICIPANTS	51](#_Toc126745735)

[23.2	PUBLICATION	51](#_Toc126745736)

[23.3	AUTHORSHIP PLAN (PRINCIPAL MANUSCRIPT)	52](#_Toc126745737)

[24	REFERENCES	52](#_Toc126745738)

[25	APPENDICES	56](#_Toc126745739)

**AGREEMENT**

This Protocol is confidential. The Investigators declare they have read this Protocol in its entirety and agree to conduct the PUMA Pilot according to the Protocol, and in accordance with the Australian NHMRC National Statement on Ethical Conduct Human Research (2018) and the ICH GCP notes for Guidance on Good Clinical Practice (CPMP/ICH/135/95).

Investigator Name:

Investigator Signature:

Date:

*Please scan and email to the PUMA Pilot PI: Luke.Perry@mh.org.au*

# 1 INVESTIGATOR LIST

**Luke Perry, MBBS(Hons), BSc**

**Primary Chief Investigator**

Research Lead, Cardiac Anaesthesia, Victorian Heart Hospital

Scientific Director, Victorian Cardiac Anaesthesia Research Laboratory, Victorian Heart Institute

Tel.: +614 7909 7205

E: [luke.perry@mh.org.au](mailto:luke.perry@mh.org.au); [luke.perry@unimelb.edu.au](mailto:luke.perry@unimelb.edu.au); [lukeaperry@gmail.com](mailto:lukeaperry@gmail.com)

**Rinaldo Bellomo AO, MBBS(Hons), MD, PhD, FRACP, FCICM**

**Chief Investigator**

Professor of Intensive Care, The University of Melbourne

Staff Intensivist and Director of Intensive Care Research, Austin Hospital

Member of Executive, Department of Critical Care, The University of Melbourne

Senior Research Advisor and Intensivist, Royal Melbourne Hospital

NHMRC Practitioner Fellow

**Julian Smith, MBBS, FRACS, FACS, MSurgEd**

**Chief Investigator**

Head, Department of Cardiothoracic Surgery, Monash Health

Professor and Head, Department of Surgery, School of Clinical Sciences, Monash University

**Reny Segal, M.B.Ch.,B., FANZCA, NBE PTEeXAM**

**Chief Investigator**

Honorary Clinical Professor, Department of Critical Care, The University of Melbourne

Head of Cardiac Anaesthesia, Department of Anaesthesia and Pain Management, Royal Melbourne Hospital

Department of Anaesthesia and Pain Management, Royal Melbourne Hospital

**Alistair Royse, MBBS, FRACS, MD, FCSANZ**

**Chief Investigator**

Senior Cardiothoracic Surgeon, Royal Melbourne Hospital

Professor of Surgery, Department of Surgery, Melbourne Medical School, The University of Melbourne

Director, Ultrasound Education Group, The University of Melbourne

**Marco Larobina, MBBS(Hons), FRACS**

**Chief Investigator**

Consultant Cardiothoracic Surgeon, Royal Melbourne Hospital

Honorary Clinical Lecturer, Department of Surgery, The University of Melbourne

Deputy Chairman, Board of Training for Cardiothoracic Surgery, Royal Australasian College of Surgeons

**Luke O’Halloran, MBBS, FANZCA**

**Chief Investigator**

Head of Cardiac Anaesthesia, Monash Medical Centre

Staff Anaesthetist, Monash Health

**Alistair McLean, BA, BSc (Hons), PhD**

**Chief Investigator / Primary Statistician**

Research Fellow in Biostatistics, Melbourne School of Population and Global Health, The University of Melbourne

**Sabine Braat, BA, MSc, MSc**

**Chief Investigator / Senior Statistician**

Senior Research Fellow in Biostatistics, Centre for Epidemiology and Biostatistics, Melbourne School of Population and Global Health, The University of Melbourne

Vice-Chair of Executive Committee, Australian Clinical Trials Alliance – Statistics in Trials Interest Group

**Lachlan Miles, MBBS(Hons), PGCertCU, PhD, FANZCA**

**Chief Investigator**

Honorary Principal Fellow and Associate Professor, Department of Critical Care, The University of Melbourne

Deputy Head of Research, Department of Anaesthesia, Austin Health

**Lisa Higgins, BPhysio(Hons), GradDipBiostat, MPH, PhD**

**Chief Investigator / Health Economist**

Critical Care Health Economist and Senior Research Fellow, Australian and New Zealand Intensive Care Research Centre, Australia

**Emily See, MBBS, BMedSci, MSc(Oxon), FRACP, FCICM, PhD**

**Chief Investigator**

Critical Care Nephrologist, Royal Melbourne Hospital

Honorary Senior Clinical Lecturer, Department of Critical Care, The University of Melbourne

**Mario Gaudino, MD, PhD, MSCE, FEBCTS, FACC, FAHA**

**Chief Investigator**

Stephen and Suzanne Weiss Professor in Cardiothoracic Surgery (II)

Assistant Dean for Clinical Trials

Professor of Clinical Epidemiology and Health Services Research at Weill Cornell Graduate School

Director of the Joint Clinical Trials Office (JCTO)

Director of Translational and Clinical Research, Department of Cardiothoracic Surgery

**Lisa Q Rong, MD, MSCE, FASE, FACC**

**Chief Investigator**

Associate Professor of Anesthesiology, Weill-Cornell Medical Center

**Jonathan Haft, MD**

**Chief Investigator**

Professor, Cardiac Surgery, Michigan Medicine

Medical Director, Michigan Medicine Cardiac Surgery

**Thomas Schwann, MD, MBA**

**Chief Investigator**

Chief of Cardiovascular Surgery, Corewell Health

**Silvana Marasco, MBBS, MSurg, MBioeth, FRACS, PhD**

**Chief Investigator**

Director of Cardiothoracic Surgery, Alfred Health

**Tim Coulson, MBBS, FANZCA, PhD**

**Chief Investigator**

Research Lead, Department of Anaesthesiology and Perioperative Medicine, Alfred Health

Staff Specialist Anaesthetist

**Jayme Bennetts, MBBS, FRACS**

**Chief Investigator**

Director of Cardiac Surgery, Victorian Heart Hospital

Professor, Cardiothoracic Surgery, Monash University

**Andrew Silvers, MBBS(Hons), FANCA**

**Chief Investigator**

Director of Anaesthesia, Victorian Heart Hospital

**Davina Taylor, PhD**

**Associate Investigator / Consumer Member**

# 2 PERSONNEL, COMMITTEES, AND RESPONSIBILITIES

## 2.1 Steering Committee

The Steering Committee will be responsible for all aspects of the PUMA Pilot. It will meet quarterly or more frequently as required.

**Chair:** Luke Perry

**Deputy Chair:** Rinaldo Bellomo AO

**Members:**

Julian Smith

Reny Segal

Marco Larobina

Alistair Royse

Luke O’Halloran

Alistair McLean

Sabine Braat

Lachlan Miles

Lisa Higgins

Davina Taylor

Emily See

Mario Gaudino

Lisa Q Rong

Jonathan Haft

Thomas Schwann

Tim Coulson

Andrew Silvers

Jayme Bennetts

Silvana Marasco

**Responsibilities:**

Overseeing all aspects of the study management including:

- Liaison with coordinating centre staff and trial management centre staff
- Overseeing funding applications
- Overseeing disbursement and administration of funds
- Ensuring fiscal responsibilities are maintained
- Development and approval of final protocol and trial materials
- Development and approval of data collection tools and methods
- General trial management issues

## 2.2 Patient Safety Committee

**Chair:** Julian Smith

**Deputy-Chair:** Reny Segal

**Members:**

Alistair Royse

Rinaldo Bellomo

Lachlan Miles

Luke O’Halloran

Marco Larobina

Luke Perry

Silvana Marasco

Mario Gaudino

Details on safety and monitoring for the PUMA Pilot, along with the responsibilities of the Patient Safety Committee, are outlined in Section 17.

## 2.3 Site Chief Investigators

**Royal Melbourne Hospital/Melbourne Private Hospital:** Prof Reny Segal

**Victorian Heart Hospital/Jessie MacPherson Private Hospital**: Prof Julian Smith

**Alfred Health:** Prof Silvana Marasco

**Austin Health:** A/Prof Lachlan Miles

**Weill-Cornell:** Prof Mario Gaudino

**University of Michigan:** Prof Jonathan Haft

**Corewell Health:** Dr Thomas Schwann

The Site Principal Investigators are responsible for the overall management of the PUMA Pilot at their individual site and in line with the study protocol:

- Screening and recruitment
- Consent
- Patient follow up
- Data collection and data transfer
- Management of data queries
- Liaison with local HREC
- Adherence to local HREC guidelines and reporting requirements

## 2.4 Additional Personnel

Research Co-Ordinators:

Amanda Hulley (Monash Health)

Shan Hung (Royal Melbourne Hospital)

Christina Kure (Alfred)

# 3 TRIAL APPROVALS AND REGISTRATION

## 3.1 TRIAL NAME

Pulmonary artery catheters for adults undergoing low risk cardiac surgery (The PUMA Pilot): a multicentre, randomised, controlled, consumer co-designed, Vanguard, pilot and feasibility clinical trial.

## 3.2 SPONSORING INSTITUTION

Melbourne Health.

## 3.3 ENDORSEMENTS

ANZCA Clinical Trials Network.

After presentations on the rationale and design of the PUMA Pilot, this trial was supported and endorsed by senior clinicians and executive members of the Departments of Cardiac Surgery, Anaesthesia, and Intensive Care at both trial sites.

## 3.4 TRIAL FUNDERS

The PUMA Pilot was successful in obtaining funding from an ANZCA Project Grant (RegKey: 23/020) and a Heart Foundation Vanguard Grant (Application ID: 106880). The Trial Funders will have no role in study design; collection, management, analysis, and interpretation of data; writing of the report; or the decision to submit the report for publication.

## 3.5 TRIAL REGISTRATION

The PUMA Pilot will be prospectively registered with the Australian and New Zealand Clinical Trials Registry (ANZCTR.org.au) after ethical approvals are obtained and before trial commencement.

## 3.6 PEER REVIEW

This Protocol underwent expert peer review by an independent clinical expert (a cardiac anaesthetist) on 23/01/2023.

The Protocol also underwent expert statistical review by the trial statisticians (CIs McLean and Braat).

As part of the grant application process, this Protocol also underwent expert peer review by 3 reviewers from ANZCA.

Review reports will be made available to the study Sponsor and HRECS.

## 3.7 HUMAN RESEARCH ETHICS COMMITTEE APPROVALS

The PUMA Pilot must receive full ethical and governance approvals from the Human Research Ethics Committees (HREC) of all sites before individual sites commence.

Each participating site will submit this protocol, and any other relevant study documentation to the responsible local or national constituted Human Research and Ethics Committee (or equivalent). Approval of the protocol, plans for obtaining consent, and related documents will be obtained prior to the start of the study at each site. It is the principal investigator’s responsibility to ensure that all conditions for approval of the study are met and that amendments to the protocol or serious adverse events are also reported to the HREC (or equivalent) as required by that committee.

# 4 LIST OF ABBREVIATIONS

| ADE | Adverse Device Effect |
| --- | --- |
| AE | Adverse Effect |
| AKI | Acute Kidney Injury |
| CVD | Cardiovascular Disease |
| eCRF | Electronic Case Report Form |
| EQ-5D-5L | EuroQOL 5 dimension, 5 level score |
| HREC | Human Research Ethics Committee |
| ICU | Intensive Care Unit |
| LOS | Length of Stay |
| MACE-30 | Major Adverse Cardiac Events at 30 days |
| MAKE-30 | Major Adverse Kidney Events at 30 days |
| MPH | Melbourne Private Hospital |
| PAC | Pulmonary Artery Catheter |
| pCRF | Paper Case Report Form |
| PICF | Patient Information and Consent Form |
| POAF | Postoperative Atrial Fibrillation |
| RCT | Randomised Controlled Trial |
| RMH | Royal Melbourne Hospital |
| RRT | Renal Replacement Therapy |
| SADE | Serious Adverse Device Effect |
| SAE | Serious Adverse Effect |
| SUSADE | Suspected Serious Adverse Device Effect |

# 5 TRIAL SUMMARIES

## 5.1 PROFESSIONAL SUMMARY

**Aim:** to establish the feasibility and acceptability of conducting a large, phase III, multi-centre, randomised controlled trial of pulmonary artery catheterisation in low risk cardiac surgery (PUMA II).

**Hypotheses:** the feasibility of proceeding to PUMA II will be established by the following criteria being met *in toto*: (1) ≥30% recruitment of eligible patients; (2) at least 80% protocol compliance; (3) complete case report form rate of ≥95%; and (5) acceptability to at least 70% of treating clinicians.

**Trial Design:** a 150 patient, investigator-initiated, consumer co-designed, multicentre, pragmatic, parallel assignment, open-label, assessor-masked, pilot and feasibility, randomised, controlled trial of pulmonary artery catheterisation versus standard care in adults undergoing low risk cardiac surgery.

**Setting:** seven tertiary academic cardiac surgery centres across Australia and the US.

**Inclusion Criteria**: adults undergoing elective coronary artery bypass grafting, isolated surgical aortic valve replacement, or surgery on the ascending aorta or aortic root with or without replacement of the aortic valve.

**Exclusion Criteria:** emergency procedures, repeat or ‘re-do’ procedure, EuroSCORE > 2% predicted 12 month mortality, pulmonary hypertension (mPAP >25 mmHg or RVSP > 35 mmHg), left ventricle ejection fraction <30%, or right heart structural abnormality.

**Intervention**: insertion of a pulmonary artery catheter pre-operatively for invasive monitoring of cardiac function during the perioperative period.

**Comparator:** insertion of a central venous access device.

**Feasibility Outcomes***:* treatment crossover, eligibility rate, recruitment rate, data collection completeness, follow-up completeness, acceptability to clinicians, and estimated resource requirement for PUMA II.

**Exploratory Outcomes:** intensity of perioperative treatment (duration and dose of inotrope and vasopressor support, SOFA score on days 1-7, net fluid balance at 24, 48, and 72 hours, use and volume of colloid fluids, peak lactate, use and dose of diuretics, transfusion of blood products, duration of mechanical ventilation, re-intubation, surgical drain output at 4 hours, return to theatre, acute kidney injury, new requirement for renal replacement therapy, post-operative atrial fibrillation, serious infection (a composite of sepsis, deep sternal wound infection, graft site infection, pneumonia, and central line associated bloodstream infection), use of transthoracic or transoesophageal echo in ICU, and insertion of alternative cardiac output monitoring devices), and clinical outcomes at discharge (discharge destination, readmission to ICU, hospital and ICU length of stay), 30 days (days spent alive and out of hospital, all-cause mortality, major adverse cardiac events, major adverse kidney events, hospital readmission), and at 90 days (all-cause mortality, change in the EuroQoL – 5 dimension – 5 level score).

**Significance:** The PUMA Pilot will provide critical feasibility data that is essential for optimising the design of the large-scale PUMA II RCT and securing Category 1 grant funding from the NHMRC. PUMA II will be the first adequately powered RCT on pulmonary artery catheters in cardiac surgery. International guidelines, expert consensus statements, and professional societies have consistently called for a trial like PUMA II. The PUMA Pilot will establish the foundations of a world-first trial that will define clinical practice guidelines and policy for the perioperative management of >2 million patients undergoing cardiac surgery every year.

**Team:** our team includes emerging investigators supported by world-renowned leaders in their fields, with an established capacity of successfully translating similar pilot trials into landmark RCTs which have attracted > $80m in funding – **CI Bellomo:** ARISE, MYRIAD, ATHOS-3, plus 24 other NEJM trials; **CI Smith**: PSP (NEJM), ATACAS (NEJM), **CI Royse**: LAAOS (NEJM), TRICS (NEJM), COMPASS (Lancet). We are supported by a Consumer Advisory Committee and expert biostatisticians. Department Executives have confirmed support for the PUMA Pilot from cardiac surgery, anaesthesia, and intensive care disciplines at both participating sites.

## 5.2 PLAIN LANGUAGE SUMMARY

**Background**

- Over 2 million people undergo heart surgery every year.
- An invasive monitoring device called a ‘pulmonary artery catheter’ can measure how well the heart pumps, and is commonly used in heart surgery patients
- Small studies suggest that pulmonary artery catheters may not benefit these patients, and may even lead to harm such as major bleeding and infection
- Some experts conclude that pulmonary artery catheters overcomplicate things and lead to unnecessary and risky treatment
- There has never been a high quality ‘randomised controlled trial’, so there is still uncertainty amongst doctors

**Aims**

- Our ultimate aim is to provide reliable evidence on whether heart surgery patients benefit from pulmonary artery catheters
- First, we must test whether a large-scale trial is feasible by starting with a small ‘pilot study’

**Methods**

- Patients undergoing low risk heart surgery will be eligible to participate
- After providing informed consent and prior to surgery, participants will be allocated at random to receive either a pulmonary artery catheter or a less invasive alternative in a 1-to-1 ratio
- We will record how smoothly the pilot study runs by collecting information on the number of patients we recruit, the number of times a person is switched from one strategy to another by their doctor, and how completely we can collect data and follow up patients
- We will also test how acceptable the study is to the treating doctors and patients, and estimate how much it would cost to do a large-scale trial

**Impact**

- If the PUMA Pilot is successful, the research team will conduct a large-scale randomised clinical trial
- This full-scale study has the potential to shield hundreds of thousands of heart surgery patients from a potentially dangerous and unnecessary monitoring device. This may lead to lower rates of complications and shorter stays in the intensive care unit, translating to better outcomes for patients and increased bed capacity for intensive care units

**Research Team**

This study will be conducted by a multidisciplinary team of academic cardiac anaesthetists,

cardiac surgeons, and intensive care specialists from the Royal Melbourne Hospital, The University of Melbourne, and Monash University. The research team includes world-renowned researchers who have a track record of completing similar pilot trials and translating them into large scale randomised clinical trials, resulting in publications in prestigious journals like the *New England Journal of Medicine* and attracting national and international funding worth over

$110 million dollars.

**Consumer Involvement**

This study is focused on helping real patients get the outcomes that matter most to them. The research team have formed a Consumer Advisory Committee of people whose lives have been affected by heart surgery to co-design this study and provide the patient perspective. The goal of this is to make our research as patient-centred as possible.

INVOLVE. Make it clear campaign. National Institute for Health Research (UK). 2021. <https://www.nihr.ac.uk/documents/plain-english-summaries/27363>.

NHMRC National Statement on Ethical Conduct in Human Research, Section 3.1.1. National Health and Medical Research Council (AU). 2018. [**https://www.nhmrc.gov.au/about-us/publications/national-statement-ethical-conduct-human-research-2007-updated-2018**](https://www.nhmrc.gov.au/about-us/publications/national-statement-ethical-conduct-human-research-2007-updated-2018)**.**

# 6 INTRODUCTION

## 6.1 BACKGROUND

### 6.1.1 Epidemiology and outcomes in cardiac surgery

Cardiovascular disease (CVD) is a leading cause of mortality, disability, and rising healthcare costs in the world.^1, 2^ The global prevalence of CVD nearly doubled to 523 million from 1990 to 2019, and now causes an estimated 18.6 million deaths and 400 million disability-adjusted life-years lost every year.^3^

Cardiac surgery is an important treatment option for several of the most prevalent and burdensome forms of CVD. Ischaemic heart disease, valvular heart disease, arrythmias and conducting system diseases, advanced heart failure, congenital heart disease, structural heart disease, cardiac tumours, and infective endocarditis, amongst others, can all be effectively treated with surgery. There is increasing demand for cardiac surgery, and it is estimated that over 2 million open heart surgeries are now performed every year by 10,000 cardiothoracic surgeons in 6,000 cardiac centres across the world.^4^ Despite increasing surgical volume overall, there are major global unmet needs in cardiac surgery.^5^ Inequality of access to cardiac surgical services leaves populations in low and middle income countries vulnerable to premature mortality from CVD.^6^

Advances in surgical technique and perioperative management have resulted in low operative mortality rates approaching 2% in Australia and the United States.^7, 8^ However, major postoperative morbidity is still experienced by up to 1 in 3 patients undergoing complex cardiac surgery.^7^ Patient factors, as well as those related to surgery, anaesthetic drugs, and cardiopulmonary bypass, can contribute to acute physiological disturbances in the perioperative period. Patients typically spend upwards of 45 hours in the intensive care unit (ICU) after cardiac surgery.^8, 9^ ICU length of stay (LOS) is an important predictor of long-term survival and quality of life.^10-12^ Accordingly, several strategies focused on enhancing recovery and reducing ICU LOS after cardiac surgery have been investigated.^13^

### 6.1.2 An abbreviated history of the pulmonary artery catheter

In 1929, Werner Forssmann inserted a catheter into his own right atrium, proving that right heart catheterisation was feasible in humans.^14^ Forssmann went on to join the Nazi Party, the Sturmabteilung (Stormtroopers, SA) and the Nationalsozialistischer Deutscher Ärztebund (Nazi Doctors' Association) in 1932.^15^ In 1945, André Cournand and Dickinson Richards measured cardiac output using right heart catheterisation.^16^ In 1956, Forssmann, Cournand, and Richards shared in the Nobel Prize for Physiology or Medicine for their work pioneering cardiac catheterisation.^17^ In 1970, Swan and Ganz published their work describing what would go on to become the modern flow-directed pulmonary artery catheter.^18^

Swan-Ganz catheters became popularised over the following 25 years. They were eventually subjected to large randomised clinical trials (RCTs). Published in 1996, the SUPPORT Trial (n = 5,735) found that pulmonary artery catheters increased mortality and resource utilisation amongst critically ill patients.^19^ The PAC-Man RCT (n = 1,041) found that PAC data altered management in 80% of patients but no difference in mortality was observed.^20^ The FACTT trial (n = 1,000) found that PAC-guided therapy did not result in improved outcomes in adults with acute lung injury, but was associated with twice as many insertion-related complications.^21^ In 2003, a multicentre trial of goal-directed therapy guided by a PAC in high-risk non-cardiac surgical patients (n = 1,994) found no evidence of benefit. The French Pulmonary Artery Catheter Study Group RCT (n = 676) on adults with shock or acute respiratory distress syndrome found no effect of PAC use on mortality.^22^ A subsequent Cochrane review of 13 RCTs involving 5,686 patients found PAC use did not affect mortality, length of stay in the hospital or ICU, or cost.^23^ The use of PACs outside the cardiac surgery population has sharply declined over the previous two decades as mounting evidence against its use translated into clinical practice.

### 6.1.3 Overview of data obtained from pulmonary artery catheters

Pulmonary artery catheters are capable of generating a range of haemodynamic data through either direct or indirect measurement.

Direct measurement:

- Central venous pressure (CVP)
- Right atrial pressure (RAP)
- Right ventricle pressure (RVP)
- Pulmonary artery pressure (PAP)
- Pulmonary artery occlusion pressure (PAOP)
- Cardiac output (CO)
- Mixed venous oxyhaemoglobin saturation (SvO_2_)

Indirect measurement:

- Systemic vascular resistance (SVR)
- Pulmonary vascular resistance (PVR)
- Cardiac index (CI)
- Stroke volume index (SVI)
- Left ventricular stroke work index (LVSWI)
- Right ventricular stroke work index (RVSWI)
- Oxygen delivery (DO2)
- Oxygen uptake (VO2)

In our clinical practice, we routinely use PACs to measure PAPs, CI/CO, and SvO_2_. ‘Wedging’ of PACs has fallen out of favour due to concerns about ischaemic, thromboembolic, and haemorrhagic complications.

### 6.1.4 Evidence for pulmonary artery catheter use: non-cardiac surgery

In 2004, a multicentre randomised controlled trial (RCT) including 1,994 high risk non-cardiac surgery patients found that PAC use had no effect on postoperative mortality (risk ratio [RR], 1.0; 95% confidence interval [CI], 0.7 to 1.3) but increased the risk of pulmonary embolism (8 cases *versus* 0, p <0.004).^24^ A subsequent Cochrane review included 13 RCTs involving 5,686 non-cardiac surgery patients also found no effect of PAC use on mortality (RR, 0.98; 95% CI, 0.96 to 1.09.^23^

### 6.1.5 Evidence for pulmonary artery catheter use: cardiac surgery

#### 6.1.5.1 Do pulmonary artery catheters save lives?

Our meta-analysis of 13 observational studies involving 2,214,900 cardiac surgery patients found no benefit on short-term mortality associated with pulmonary artery catheter use (pooled odds ratio (OR), 1.15; 95% confidence interval (CI), 0.93 - 1.59) (Figure 1) (**CIA** *and* **CIB** *personal communication*)*.

Figure 1: Forest Plot for 30-day mortality from our meta-analysis.

We subsequently conducted a retrospective cohort study with propensity score matching of 5,614 patients using a comprehensive Harvard database. In this study, pulmonary artery catheterisation was not associated with either short-term (OR, 1.80; 95% CI, 0.83 – 3.92) or long-term (hazards ratio (HR), 1.18; 95% CI 0.86 – 1.61) mortality (Figure 2).

Figure 2: Kaplan-Meier curve for long-term survival from the PUMA Cohort Study.

Moreover, our analysis of 1,442,293 patients using the US National Inpatient Sample found PAC use was associated with increased mortality (OR, 1.21; 95% CI, 1.13 – 1.39) (**CIA** *personal communication*)*. However, observational studies are prone to bias resulting from unmeasured confounding, and a mortality benefit cannot be excluded without a rigorous RCT.^25^

#### 6.1.5.2 A number needed to treat? Pulmonary artery catheters, over-intervention, and patient harm

Pulmonary artery catheters are a monitoring device, not an intervention, and therefore have little direct influence on patient outcome. Instead, the data they generate are used to guide therapeutic decision-making in the perioperative period. It has been suggested that these data may unintentionally lead to patient harm by triggering unnecessary and dangerous interventions and causing hesitancy to discharge patients from the Intensive Care Unit (ICU).^26, 27^ This theory is supported by several large observational studies that have shown an association of PAC use with higher intensity perioperative treatment including prolonged inotrope use^28-30^, positive fluid balance^28^, duration of mechanical ventilation^28, 31^, blood transfusion^32^, and prolonged ICU length of stay (LOS).^28, 32, 33^ This pattern is corroborated by our propensity-matched analysis of 5,614 patients which found that PAC use was associated with inotrope use for >4 hours (OR, 3.78; 95% CI 3.31 to 4.32), packed red cell transfusion (OR, 2.03; 95% CI, 1.92 – 2.14), serious infection (OR, 1.32; 95% CI, 1.20 – 1.48), acute kidney injury (OR, 1.41; 95% CI, 1.29 - 1.52), and prolonged ICU length of stay (LOS) (adjusted mean difference, 16 hours; 95% CI, 12.2 - 19.9 hours) (**CIA** *and* **CIB** *personal communication*)*. Our meta-analysis on ICU LOS indicated PAC use was associated with a statistically and clinically significant increased ICU LOS (pooled mean difference [hours], +15.03; 95% CI, 6.26 – 23.81) (Figure 3).

Figure 3: Forest plot for ICU LOS from our meta-analysis.

In addition to being associated with higher intensity perioperative treatment and perioperative complications, PACs are also capable of causing rare but catastrophic iatrogenic harm in 0.1 to 3% of cases. ^34, 35^ These complications include pulmonary artery rupture, endobronchial haemorrhage, right ventricular perforation, pulmonary infarction, and life-threatening arrythmias.

#### 6.1.5.3 PAC data is subject to error and its clinical relevance has been disputed

Estimates of cardiac output (CO) derived from PACs are subject to error related to technique, miscalculation, cardiac abnormalities, patient posture, and extracardiac abnormalities. The accuracy and clinical relevance of these data have been questioned.^26, 36, 37^ One review observed that cardiac output must change by up to 25% in order to be detected by a PAC.^27^

#### 6.1.5.4 Global practice is highly varied and is suggestive of genuine equipoise

A large survey by the Society of Cardiovascular Anaesthesiologists assessed PAC usage by cardiac anaesthetists in the United States, Canada, Europe, and Australia.^38^ Two-thirds of respondents reported using a PAC in at least 75% of cases involving cardiopulmonary bypass. Moreover, national surveys from Germany, Austria, and Italy underscored heterogeneity in practice, with PAC rates of 47.5%, 33%, and 18.3%, respectively. ^39-41^ Clinicians from developing countries have also described significant variation in their use of PACs.^42^ Our analysis of Australian Medicare Benefits Scheme data from 2016-2021 confirmed substantial regional variations in practice within the Australian private healthcare setting.^43^ In this analysis, PAC use was highest in Western Australia (81%), Victoria (78%) and New South Wales (64%), and lowest in Queensland (4.6%) and South Australia (7%) (Figure 4) (**CIA** *and* **CIB** *personal communication*)*.

Figure 4: Regional heatmap of PAC rates in the Australia private healthcare setting from our analysis of MBS data 2013-2023.

#### 6.1.5.5 International guidelines acknowledge the urgent need for a randomised trial

Several international society guidelines have made weak recommendations (Class IIb) based on low quality evidence (Level B and C) that PACs should not be routinely used in *all* cases, and that RCTs are needed to identify the safety and effectiveness of PACs across different risk groups.^44-46^ Our literature review identified 3 small trials evaluating PAC use in cardiac surgery (**CIA** *and* **CIB** *personal communication*)* . Two trials involving 40 and 58 patients compared goal-directed treatment using a PAC with experimental devices (bioreactance or pulse contour based).^47, 48^ The third trial, conducted over 30 years ago, enrolled 226 patients into 5 groups, had a 65% protocol violation rate, and focused on economic outcomes.^49^ These trials lack external validity to current practice and there is an urgent and unmet need for an appropriately powered, well designed RCT.

In 2003, the American Society of Anaesthesiologists Task Force on Pulmonary Artery Catheterisation concluded that pulmonary artery catheters have no role in routine perioperative management, but that a specific subpopulation for which the use of this device may be beneficial cannot be excluded.^44^ In 2013, the Choosing Wisely organisation highlighted that PAC use in low risk cardiac surgery is likely to represent low value care.^50^ Clinical uncertainty in low, moderate, and even high risk cases has been highlighted by several reviews, expert opinions, and editorials. These articles have consistently called for an RCT on the topic.^27, 36, 51-53^

#### 6.1.5.6 Rationale for the PUMA Pilot

Given that:

1. Observational data suggests PAC use is an independent predictor of higher intensity perioperative treatment and worse outcomes
2. International guidelines have highlighted the lack rigorous evidence on PACs in cardiac surgery and were unable to make firm recommendations on their use
3. There is widespread variation in practice internationally (a marker of clinical uncertainty)
4. There has never been an adequately powered RCT evaluating PAC use in cardiac surgery
5. It is essential to conduct a pilot and feasibility study before conducting a large definitive RCT
6. Our team has established capacity to translate pilot and feasibility trials into large NHMRC-funded RCTs that are published in high-impact journals and transform practice globally.

We propose a 150 patient randomised controlled pilot and feasibility trial of pulmonary artery catheterisation in adults undergoing low risk cardiac surgery.

#### 6.1.5.7 Outcomes and significance

This research will address a highly editorialised, contentious, and important area of clinical uncertainty affecting over 2 million cardiac surgery patients annually. Contemporary debates have fiercely highlighted the urgent and unmet need for a definitive trial like PUMA II. ^27, 36, 51-53^ The PUMA Pilot will provide critical feasibility data that is essential for optimising the design of the large-scale PUMA II RCT and securing Category 1 grant funding from the NHMRC or MRFF.

PUMA II will be the first adequately powered RCT on pulmonary artery catheters in cardiac surgery. It will provide a definitive answer to this question for a vulnerable group of patients and drastically influence perioperative practices by providing critical data to support the de-implementation of low-value care. PUMA II has the ability to transform global practice and define international clinical guidelines. If PUMA II confirms that PACs increase the risk of complications and ICU LOS without conferring a survival benefit, the results could be immediately translated into clinical practice. The resulting public health and economic implications would be substantial and widespread. We hope to align local and international practice, which is currently variable, not evidence-based, and potentially low-value care, with updated international guidelines informed by high quality evidence. By simplifying care and shielding patients from unintended harm related to a potentially unnecessary and invasive monitoring device, PUMA II has the potential to improve the cardiovascular health of Australians and prevent death and disability in millions of patients globally every year.

# 7 OBJECTIVES

## 7.1 Aim

The PUMA Pilot aims to establish the feasibility of conducting a large Phase III randomised controlled trial of pulmonary artery catheterisation for low-risk cardiac surgery (PUMA II).

## 7.2 Hypothesis

The feasibility of proceeding to PUMA II will be established by the following criteria being satisfied *in toto*:

1. At least 30% of eligible patients are enrolled and undergo randomisation
2. At least 80% of participants receive the assigned intervention and do not ‘cross-over’
3. At least 95% of case report forms are complete

# 8 TRIAL DESIGN

A 150 patient, multicentre, prospective, randomised, open-label, blinded endpoint (PROBE), pragmatic, consumer co-designed, pilot and feasibility trial of pulmonary artery catheterisation in adults undergoing low-risk cardiac surgery.

## 8.1 SETTING

The PUMA Pilot will be conducted at the Royal Melbourne Hospital (RMH; the Trial Co-Ordinating Centre), Victorian Heart Hospital, Weill-Cornell Medical Center, University of Michigan Cardiovascular Center, Alfred Health, Austin Health, and Corewell Health, seven University-affiliated tertiary referral centres in Australia and the US. Some patients who undergo surgery at RMH may spend their immediate postoperative ICU stay at the Melbourne Private Hospital (MPH) under a current public-in-private arrangement. Some patients who undergo surgery at VHH may similarly be managed under a private-in-public arrangement through Jessie MacPherson Private Hospital. Follow-up will take place by telephone or inpatient visits if appropriate on postoperative days 30 and 90.

## 8.2 PILOT NATURE OF THIS STUDY

The PUMA Pilot aims to confirm or deny the feasibility of conducting a large-scale RCT (PUMA II) and identify modifiable barriers to feasibility and acceptability before proceeding. A pilot trial is a type of feasibility study designed to test the feasibility of methods and procedures for later use in a large scale RCT.^54^ Pilot trials in anaesthesia and critical care are increasingly being recognised as essential precursors to conducting quality RCTs.^55^ Achieving pre-defined feasibility endpoints in a pilot study is vital to the success of a subsequent large scale RCT where a similar trial has never been conducted. The PUMA Pilot is not designed to perform hypothesis-testing statistical analyses of clinical outcomes, and such analyses will be conducted and reported with caution.

## 8.3 TRIAL TIMELINE

Recruitment will commence in July 2024 and is expected to be completed by early 2025, with 90 day follow-up of the final patient expected to be completed in March 2025.

## 8.4 TRIAL FLOWCHART

Figure 5: PUMA Pilot flowchart.

## 8.5 DEFINING THE PATIENT JOURNEY

The PUMA Pilot is pragmatic by design, is ‘hands-off’ during the surgical admission, and involves minimal patient contact and therefore administrative burden. There will be 3 main points of contact with study personnel: (1) At the cardiac surgery pre-admission clinic where patients will be invited to participate, provide informed consent, and complete a 3 minute quality of life questionnaire; (2) at 30-days post-op for a telephone review to collect outcomes data; (3) at 90-days post-op for a telephone review to collect outcomes data and repeat the quality of life questionnaire (Figure 6). Aside from the initial strategy of pulmonary artery catheter *versus* central venous catheter, all aspects of patient management are at the sole discretion of the treating clinicians. No other additional to standard care contact, treatments, or investigations are required.

Figure 6: Patient Journey for the PUMA Pilot.

# 9 TRIAL POPULATION

## 9.1 NUMBER OF PARTICIPANTS

We aim to enrol 150 eligible patients (75 in each group) into the PUMA Pilot using similar procedures successfully implemented by the Chief Investigators for other large randomised controlled trials.

## 9.2 INCLUSION CRITERIA

1. Undergoing coronary artery bypass grafting, isolated surgical aortic valve replacement or repair, or surgery on the ascending aorta or aortic root with or without replacement/repair of the aortic valve.
2. Age > 18 years old

## 9.3 EXCLUSION CRITERIA

1. Emergency procedures where surgery must be performed within 24 hours of the decision to operate (or before the start of the next business day)
2. Repeat or ‘re-do’ procedures
3. EuroSCORE II >2%^56^
4. Pulmonary hypertension defined by right ventricular systolic pressure > 35 mmHg or a mean pulmonary artery pressure on right heart study of >25 mmHg
5. Any degree of right ventricle systolic dysfunction as identified by cardiology report on most recent preoperative transthoracic echocardiogram. May be identified by cardiologist reported right ventricular systolic dysfunction, TAPSE < 15mm, or RVFAC < 35%.
6. Severe left ventricular systolic impairment (ejection fraction <30%)
7. Right-heart structural abnormality (e.g. severe tricuspid or pulmonary stenosis or regurgitation, tumour, atrial or ventricular septal defect)

## 9.4 CO-ENROLMENT

Due to the pilot and randomised nature of the PUMA Pilot, patients will be allowed to co-enrol in other randomised and observational studies if there are no safety concerns raised by the CIs of both study and the patient does not feel overly burdened by participation.

# 10 PARTICIPANT SELECTION AND ENROLMENT

## 10.1 SCREENING

Patients on the elective cardiac surgery waitlist will be screened for eligibility by trained research nurses using a simple eligibility criteria screening tool (Appendix). Additional potentially eligible patients who are scheduled to undergo inpatient surgery and who meet all the inclusion and none of the exclusion criteria will be identified from the cardiac surgery theatre booking systems. These patients will be approached on the ward at a convenient time and after agreement to initial contact is confirmed by a member from the patient’s clinical team. A screening log (Appendix) will be maintained by study personnel in accordance with the Data Management Plan.

## 10.2 INFORMED CONSENT

### 10.2.1 Process

For patients on the elective cardiac surgery waitlist, initial contact will be made by telephone at a convenient time and before their routine pre-admission clinic appointment. The purpose of this initial phone contact is to provide an overview of the study, introduce the possibility of involvement, and allow the patient the opportunity to decline further in-person discussion at their pre-admission appointment. This should help reduce the added stress and burden of additional contact at pre-admission clinic in patients who are definitely not interested. Eligible patients demonstrating initial interest from the telephone call will then be invited to participate in-person by a trained research nurse or a study investigator on the day of their routine pre-admission clinic visit.

At the preadmission clinical appointment, eligible patients will be reminded of the possibility for study participation. The study rationale, as well as the potential benefits and risks of participation will be communicated to the patient, and the patient will receive an HREC-approved patient information and consent form (PICF) in plain language. The patient will have the opportunity to ask any questions they may have. Written informed consent will be obtained from all eligible patients willing to participate, with appropriate support personnel and interpreter services made available. Patients will be reminded of their right to withdraw from the study for any reason and at any time without their care or therapeutic relationships being adversely affected. The signed consent form will be stored securely in a locked file in a secure office. Once informed consent is obtained, patients will be allocated a unique research study ID. On the day of enrolment, the patient will undergo a voluntary quality of life questionnaire (EQ-5D-5L) by a trained research nurse.^57^ The EQ-5D-5L instrument (Appendix) is cognitively undemanding, and takes only a few minutes to complete. The treating cardiac anaesthetist and cardiac surgeon will be notified of their patient’s enrolment in the study.

All interaction between research staff and potential or actual participants and their legal surrogate will take into consideration the stress or emotional factors associated with undergoing cardiac surgery and ensure that the dependency of potential participants and their relatives on medical personnel providing treatment does not compromise the freedom of decision making to participate (as per the NHMRC National Statement on the Ethical Conduct of Research in Humans 2007 (Updated 2018) 4.4.11).

The multi-staged approach to recruitment (initial contact by phone, then an in-person discussion days later at pre-admission clinic) allows participants multiple opportunities to consider their involvement in the study, to ask questions, and to avoid feeling rushed or coerced before providing informed consent.

Privacy: potential participants are entitled to privacy and all face-to-face interactions with research personnel will be conducted in a private clinic room.

Potential impacts of existing relationships on recruitment: it is possible that some of the patients will be treated clinically by cardiac surgeons, intensivists, and anaesthetists who are also investigators on the PUMA Pilot. To mitigate the potential impact of this, the research will be discussed with the potential participant by chief investigators and experienced senior research nurses who will not be involved in the patients' clinical care. Patients will be reminded that their decision to participate or not to participate will in no way affect their therapeutic relationships.

Training and experience of recruiters: participants will be recruited by trained experienced research nurses and chief investigators. All trial personnel involved in recruitment must have up-to-date Good Clinical Practice Training and be familiar with the National Statement.

### 10.2.2 Responsibilities

It is the responsibility of the Chief Investigators and the trained Research Nurses to confirm eligibility and that informed consent is obtained according to local procedures and legislation. The CI’s are responsible for ensuring the informed consent form (ICF) is signed and dated by all parties and that it is securely transferred to the PUMA Pilot trial office before randomisation is conducted. All research staff are responsible for conducting the trial to high ethical standards, prioritising respect for patients, and following all relevant local laws, policies, and HREC requirements.

The CIs are responsible for ensuring the patient receives the ICF, PICF, and contact details of the trial manager.

### 10.2.3 Documentation

The patients and a senior member of the research team should sign and date the ICF. Hardcopies of the ICF will be given to the patient, stored in their medical record, and transferred to the Puma Pilot trial office to be securely stored. An electronic copy of the signed ICF will be uploaded into the patient’s electronic case report form (eCRF).

# 11 INTERVENTIONS

## 11.1 PULMONARY ARTERY CATHETERS

Patients randomised to receive a pulmonary artery catheter will receive the intervention according to local procedures. At both the Royal Melbourne Hospital and Monash Health, standard procedure involves insertion of an Edwards Swan-Ganz catheter (3 lumen, 7.5 Fr) through an Arrow Multi-lumen Access Catheter (MAC) sheath (9 or 8.5 Fr), although the specific type of pulmonary artery catheter and method of insertion is at the discretion of the treating consultant cardiac anaesthetist. We place no restriction on how data derived from the pulmonary artery catheter is to be used by the treating clinicians (i.e. the study is pragmatic by design and does not include a protocol for goal-directed therapy).

Functional Description (from the manufacturer): Swan-Ganz thermodilution catheters provide diagnostic information to rapidly determine haemodynamic pressures and cardiac output when used with a compatible cardiac output computer. It has a 7.5 French size catheter body and 110cm usable length, diameter of the inflated balloon is 13 mm. It has a thermistor and 4 lumens. In addition to the standard distal and injectate lumens, the Swan-Ganz VIP thermodilution catheter has an additional lumen that provides direct access to the right atrium. Intended Purpose Swan-Ganz thermodilution catheters are indicated for the assessment of a patient's haemodynamic condition through direct intracardiac and pulmonary artery pressure monitoring, cardiac output determination, and for infusing solutions. The distal (pulmonary artery) port also allows sampling of mixed venous blood for the assessment of oxygen transport balance and the calculation of derived parameters such as oxygen consumption, oxygen utilisation coefficient, and intrapulmonary shunt fraction.

## 11.2 COMPARATOR

Patients in the control group will receive a central venous access device (CVAD) with direct central venous pressure monitoring. The CVAD will be inserted through a MAC sheath. The site and specific type of CVAD is at the discretion of the treating anaesthetist. All aspects of perioperative patient management will be at the discretion of the treating team. The treating clinicians can decide at any time to insert a pulmonary artery catheter if determined to be clinically indicated. We place no restriction on the use of other means of haemodynamic monitoring (e.g. transthoracic or transoesophageal echocardiography or pulse index continuous cardiac output (PiCCO) monitoring).

**All aspects of participants’ perioperative management are at the discretion of the treating clinicians.**

# 12 OUTCOMES

## 12.1 FEASIBILITY OUTCOMES

### 12.1.1 Primary feasibility outcome

| **Protocol compliance** | **Definition:** the proportion of participants receive their assigned intervention and do not ‘cross-over’ (e.g. receive a PAC if assigned to the control group).  **Timing:** after randomisation occurs until first discharge from the intensive care unit.  **Minimum target:** 80%. |
| --- | --- |

### 12.1.2 Secondary feasibility outcomes

| **Eligibility rate** | **Definition:** the proportion of patients booked for cardiac surgery meeting all of the inclusion criteria and none of the exclusion criteria.  **Minimum target:** 30%. |
| --- | --- |
| **Recruitment proportion** | **Definition:** the proportion of eligible patients who provide written informed consent and undergo randomisation.  **Minimum target:** 30%. |
| **Recruitment rate** | **Definition:** the number of participants recruited per site per 30 days.  **Minimum target:** 8 patients/site/30 days. |
| **Complete case report form proportion** | **Definition:** the proportion of participants with complete paper and electronic case report forms.  **Minimum target:** 95% |
| **Acceptability to clinicians** | **Definition:** general willingness to have their patients enrolled in the PUMA Pilot ascertained indirectly as the proportion of eligible patients whose participation was not progressed due to a lack of equipoise from the surgeon or anaesthetist. |
| **Estimated resource expenditure** | **Definition:** the estimated cost, in Australian dollars, of all costs incurred in the conduct of the PUMA Pilot. Used to inform funding requirements for the subsequent large-scale PUMA II Trial.  **Timing:** from trial planning phase until the end of the trial (see ‘End of Trial’ defined in Section 20). |

## 12.2 PILOT OUTCOMES

As this is a pilot study, our aim is not to perform hypothesis-testing statistical analyses on clinical outcomes. However, we will record the following clinical outcomes in order to closely simulate the data collection procedures of the planned PUMA II trial and to inform its sample size calculations.

#### 12.2.1.1 ICU Outcomes

| **Time in postoperative organ dysfunction (T_POD_) at 30 days^1^** | **Definition:** the combined duration of requirement of one or more of the following life-sustaining therapies: mechanical ventilation (not including non-invasive ventilation), vasopressor or inotrope therapy, mechanical circulatory support (e.g. extracorporeal membrane oxygenation or intra-aortic balloon pump), and continuous renal replacement therapy or new intermittent hemodialysis.  **Timing**: from admission to ICU until 30 days. |
| --- | --- |
| **ICU Length of Stay** | **Definition:** the number of hours from the index admission to ICU until the patient is discharged from the ICU |
| **SOFA Score** | **Definition:** the Sequential Organ Failure Assessment (SOFA) score calculated using validated methods.^2^  **Timing:** daily from admission to ICU until 7 days. |
| **Duration of vasopressor or inotropic support** | **Definition:** the total number of hours a patient is administered vasopressor and/or inotropic support. Vasopressor support is defined by any use of: norepinephrine, vasopressin, metaraminol, or phenylephrine. Inotrope support is defined by any use of: epinephrine, dobutamine, dopamine, levosimendan, milrinone, or isoprenaline.  **Timing**: from admission to ICU until 7 days. |
| **Cumulative dose of vasoactive drugs** | Definition: Cumulative dose of vasopressor drugs, measured in noradrenaline equivalents using established methods, ^3^ and inotropic drugs.  **Timing**: from admission to ICU until 7 days. |
| **Cumulative dose of diuretic drugs** | **Definition:** cumulative dose (in mg) of frusemide administered orally or intravenously.  **Timing:** from admission to ICU until 7 days. |
| **Cumulative dose of albumin** | **Definition:** cumulative dose (in g) of albumin administered.  **Timing:** from admission to ICU until 7 days. |
| **Net fluid balance at 24, 48, and 72 hours** | **Definition:** the cumulative volume of fluids administered to the patient from any source minus the cumulative measured volume of fluids removed from the patient by any source.  **Timing:** from admission to ICU to 24, 48, and 72 hours. |
| **Peak lactate at 24, 48, and 72 hours** | **Definition:** the highest value recorded of plasma lactate levels in mmol/L.  **Timing:** from admission to ICU to 24, 48, and 72 hours. |
| **Return to theatre** | **Definition:** return to theatre for any cardiothoracic reoperation.  **Timing:** from admission to ICU until 7 days. |
| **Duration of mechanical ventilation** | **Definition:** the number of hours from when the patient is first placed on mechanical ventilation in the operating theatre until final extubation (separate periods of intubation and mechanical ventilation are additive).  **Timing**: during the index ICU admission. |
| **Re-intubation** | **Definition**: re-insertion of an endotracheal tube after one is removed.  **Timing**: during the index ICU admission. |
| **Renal replacement therapy** | **Definition:** the patient receives any form of renal replacement therapy which was not required preoperatively.  **Timing:** from admission to the ICU until 30 days have elapsed. |
| **Utilisation of echocardiography** | **Definition:** the number of times each of the following are performed: bedside transthoracic echocardiography (TTE), formal diagnostic TTE performed by a sonographer or cardiologist, and transesophageal echocardiography.  **Timing:** from admission to ICU until 7 days. |
| **Use of non-invasive cardiac output monitoring devices** | **Definition:** use of any non-invasive cardiac output monitoring device (e.g. pulse contour devices [PiCCO], bioreactance devices, etc.).  **Timing:** from admission to ICU until 7 days. |
| **Readmission to the ICU** | **Definition:** the patient is readmitted to the ICU after they have been discharged and transferred to another setting.  **Timing:** from discharge from ICU until the patient is discharged from hospital. |

#### 12.2.1.2 Hospital Outcomes

| **Blood product transfusion** | **Definition:** the volume in milliliters transfused of each of the following blood products: packed red blood cells, platelets, cryoprecipitate, fresh frozen plasma, and prothrombin complex concentrates.  **Timing**: from start of surgery until hospital discharge |
| --- | --- |
| **Serious infection** | **Definition:** a composite of sepsis, deep sternal wound infection, graft site infection, radiographically and clinically proven pneumonia, and central-line associated blood stream infection (CLABSI)  **Timing:** from admission to the ICU until discharge from hospital. |
| **Post-operative atrial fibrillation** | **Definition:** the development of new-onset and persistent (lasting at least 60 minutes or requiring any intervention) atrial fibrillation from admission to ICU until 7 days have elapsed. |
| **Acute kidney injury** | **Definition**: acute kidney injury, defined by KDIGO Criteria, occurring during the hospital admission^4^  **Timing:** from induction of anesthesia until discharge from hospital |
| **In-hospital mortality** | **Definition:** death due to any cause occurring during the index hospital admission  **Timing:** from induction of anesthesia until discharge from hospital |
| **Hospital length of stay** | **Definition:** the number of hours from admission to ICU until the patient is discharged from hospital. |
| **Discharge destination** | **Definition:** the type of place the patient is discharged to. Categories include: rehabilitation facility/step-down care, another hospital, the patient’s home, supported accommodation, nursing facility, or other. |

#### 12.2.1.3 30-Day Outcomes

| **Days alive and out of hospital at 30 days** | **Definition:** the number of days from admission to ICU until 30 days have elapsed or the patient dies, minus the number of days the patient spent admitted to any hospital during this period (either during the index hospitalization or any subsequent readmission.)  **Timing:** from admission to ICU until 30 days. |
| --- | --- |
| **All-cause mortality** | **Definition:** death due to any cause.  **Timing:** from the start of anesthetic care until 30 days have elapsed. |
|  |  |
| **Hospital readmission** | **Definition**: the patient is readmitted to any hospital for any reason.  **Timing:** from admission to ICU until 30 days have elapsed. |
| **Major Adverse Cardiac Events (MACE-30)** | **Definition**: a composite of all-cause mortality, stroke, AMI, repeat revascularization procedure, and readmission to hospital due to heart failure  **Timing:** from admission to the intensive care unit until 30 days have elapsed. |
| **Major Adverse Kidney Events (MAKE-30)^5^** | **Definition:** a composite of all-cause mortality, need for renal replacement therapy, and persistent renal dysfunction^5^  **Timing:** from admission to the intensive care unit until 30 days have elapsed. |

#### 12.2.1.4 90-Day Outcomes

| **All-cause mortality** | **Definition:** death due to any cause.  **Timing:** from the start of anesthetic care until 90 days have elapsed. |
| --- | --- |
| **Change in quality of life** | **Definition:** EuroQoL – 5 dimension – 5 level (EQ-5D-5L) score at 90-days less the EQ-5D-5L score at enrolment.^6^  **Timing:** assessed at enrolment and at 90 days by telephone interview. |
| **Days alive and out of hospital at 90 days** | **Definition:** the number of days from admission to ICU until 90 days have elapsed or the patient dies, minus the number of days the patient spent admitted to any hospital during this period (either during the index hospitalization or any subsequent readmission.)  **Timing:** from admission to ICU until 90 days. |

# 13 ASSIGNMENT OF INTERVENTIONS

## 13.1 ALLOCATION SEQUENCE GENERATION

Patients will be randomly allocated (1:1) to receive either a pulmonary artery catheter or a central venous access device after induction of anaesthesia. Randomisation will be through a centralised, secure, web-based platform using randomly permuted blocks stratified by trial site and surgical urgency. The randomisation table will be generated by an independent statistician, who will not reveal the block size or allocation sequence to the trial personnel, patients, and treating clinicians to ensure allocation concealment. Randomisation will occur no more than 24 hours before the scheduled surgery to avoid bias resulting from unexpected cancellations.

## 13.2 ALLOCATION CONCEALMENT

Due to the use of an independent statistician for random sequence generation, trial personnel, patients, and treating clinicians will be unable to determine or predict treatment allocation until after the randomisation form is submitted by study personnel.

## 13.3 IMPLEMENTATION OF TREATMENT ALLOCATION

Once a patient is enrolled, undergoes randomisation, and is allocated an intervention, study personnel will contact the treating cardiac anaesthetist and surgeon informing them of their patients treatment allocation and providing them with details on how to report adverse events and provide feedback to the Chief Investigators.

## 13.4 BLINDING

Due to the nature of the intervention, blinding of patients and their treating team is not possible. All other personnel including outcome assessors and trial statisticians will be blinded to participants’ treatment allocation until the database has been cleaned and pre-processed for analysis.

## 13.5 CROSSOVER EVENTS

The treating consultant anaesthetist or intensivist may at any time choose to insert or remove a pulmonary artery catheter if it is determined to be clinically indicated. If crossover occurs, reasons for this will be documented in the pCRF and transcribed into the eCRF.

# 14 WITHDRAWAL OF PARTICIPANTS

Participants have the right to withdraw from the PUMA Pilot for any reason and at any time. Participants may also be withdrawn by the CI’s, treating doctor, or Sponsor if there are concerns for patient safety or for administrative reasons. In the event of withdrawal, the primary reason will be documented in the pCRF and eCRF. Withdrawal can involve either all levels of participation or only certain levels of participation (e.g. patients can decline 30 and 90 day follow-up but still wish to participate in the inpatient reviews).

# 15 TRIAL PROCEDURES

## 15.1 Summary

|  | Up to 3 months pre-op | Pre-admission clinic visit | Pre-operative | Day of Surgery | ICU discharge | Hospital discharge | Post-op day 30: telephone review | Post-op day 90: telephone review |
| --- | --- | --- | --- | --- | --- | --- | --- | --- |
| Screening | X |  |  |  |  |  |  |  |
| Consent |  | X |  |  |  |  |  |  |
| Enrolment |  | X |  |  |  |  |  |  |
| EuroQOL-5d-5l |  | X |  |  |  |  |  | X |
| Demographic data recorded |  |  | X |  |  |  |  |  |
| Medical comorbidities recorded |  |  | X |  |  |  |  |  |
| Laboratory results recorded |  |  | X |  |  |  |  |  |
| Echocardiography results recorded |  |  | X |  |  |  |  |  |
| Coronary angiography results recorded |  |  | X |  |  |  |  |  |
| Immediate adverse events recorded |  |  |  | X |  |  |  |  |
| Operative details recorded |  |  |  | X |  |  |  |  |
| ICU outcomes |  |  |  |  | X |  |  |  |
| Hospital outcomes |  |  |  |  |  | X |  |  |
| Post-discharge outcomes |  |  |  |  |  |  | X | X |

## 15.2 Data Collection and Patient Observations

Both paper and electronic case report forms will be started as soon as possible after patient enrolment and will be maintained as up to date throughout the course of the patients participation in the study. Random compliance checks will be performed by Chief Investigators to ensure the study protocols are being followed. See the CRF in the Appendix for the complete list of data to be captured.

## 15.3 Pre-operative period

The following patient data will be recorded into the CRF’s. See the attached CRF for specifics and guidance.

Demographic data: age, sex, height, weight, ethnicity, primary place of residence (home independent, home with some dependence, supported accommodation, or high-level care nursing facility).

Medical and surgical co-morbidities: the patients complete medical and cardiac surgical history.

Cardiac investigations: results from pre-operative electrocardiograms, transthoracic echocardiograms, and coronary investigations (angiogram or CT coronary angiogram).

Laboratory investigations: all pre-operative laboratory results will be recorded, including but not limited to haemoglobin concentration, white cell counts, and platelet concentration, renal function and electrolyte counts, cardiac biomarkers (troponin and B-type natriuretic peptide), iron studies, and coagulation profile.

## 15.4 Day of Surgery

Operative details including final surgery performed, cross-clamp time, total cardiopulmonary bypass time.

Immediate complications related to PAC insertion documented by the treating anaesthetist.

## 15.5 Post-operative Day 1

Wound review: as part of routine care, the treating cardiac surgeon or their delegate will document the results of their review of the sternotomy and vascular harvest wounds for the presence of infection.

## 15.6 Post-operative Day 3

Wound review: the treating cardiac surgeon or their delegate will document the results of their review of the sternotomy and vascular harvest wounds for the presence of infection as part of routine care.

## 15.7 ICU Discharge

Date and time of PAC/CVAD removal

ICU Outcomes (Section 3.4.2) will be adjudicated by study personnel and documented in the CRF.

## 15.8 Hospital Discharge

Hospital outcomes (Section 3.4.2) will be adjudicated by study personnel and documented in the CRF.

## 15.9 Post-operative Day 30

Telephone interview (or in person review if still admitted) to ascertain: vital status, readmission to hospital, quality of life score EQ-5D-5L.

Medical record and laboratory result review for the 30-day outcomes.

## 15.10 Post-operative day 90

Telephone interview (or in person review if still admitted) to ascertain: vital status, DAH, quality of life score EQ-5D-5L.

# 16 DATA MANAGEMENT PLAN

## 16.1 Processes

The Chief Investigators are responsible for ensuring data management protects the safety, rights, welfare, and privacy of research participants and the wider community. This Data Management Plan (DMP) was designed in accordance with the RMH Data Management in Research Guideline

After randomisation, patients will be allocated a unique study number. The site research coordinator will compile an enrolment log including the patient’s name, date of birth, hospital identification number, unique study number and date and time of randomisation. Subsequent data will be identified by the unique study number only. The enrolment log and study data will be kept separately. Study data will be entered into a password protected REDCap database managed by the co-ordinating centre. Participants will be followed up until death or 90 days. No identifying data will be entered into the database.

Similarly, hard copies of enrolment log and study data collected on paper CRF’s will be stored separately in the locked office of the principal investigator or research coordinator. Only the research team will have access to this information, and they will not disclose this information to any other person or entity.

When archiving, the study site investigators will take all appropriate measures to safeguard and prevent access to this data by any unauthorised third party.

The investigator will maintain the confidentiality of all study documentation and take measures to prevent the accidental or premature destruction of these documents. The investigator will retain the study documents at least 15 years after the completion or discontinuation of the study. The investigator must notify the study management committee prior to destroying any essential study documents following the study completion or discontinuation.

If any investigator retires, relocates, or otherwise withdraws from conducting a study, the responsibility for maintaining records may be transferred to the coordinating centre or another investigator. The coordinating centre must be notified of and agree to the change. All associated documentation must also be updated.

## 16.2 Data Variables Collected

### 16.2.1 Baseline Data

- Age
- Sex
- Ethnicity
- Height
- Weight
- BMI
- Primary place of residence (type of accommodation)
- EuroQoL-5d-5l (day of consent)

### 16.2.2 Comorbidities

- Hypertension
- Dyslipidaemia
- Smoking history
- COPD
- Diabetes
- Atrial fibrillation
- Stroke
- Peripheral vascular disease

### 16.2.3 Regular medications

- Angiotensin-converting enzyme inhibitor
- Angiotensin receptor blockers
- Antiplatelets
- Anticoagulants
- Bet-blockers
- Diuretics
- Oral hypoglycaemics
- Insulin
- Statins

### 16.2.4 Surgical details

- Surgery performed
- Date/time of surgery
- Cardiopulmonary bypass time
- Aortic cross clamp time
- Estimated blood loss

### 16.2.5 Perioperative data

- EuroSCORE
- ASA
- Baseline creatinine level
- Baseline haemoglobin level
- Chest tube drainage in first 24 hours

### 16.2.6 Line data

- Assigned intervention received (PAC or CVAD)
- Date/time of insertion
- Complications of insertion
- Date/time of removal
- Any documented complications associated with use

### 16.2.7 ICU Data

- Date/times of ICU admission and discharge
- Readmission
- Return to theatre
- Postoperative atrial fibrillation
- Mechanical ventilation
  - Duration of mechanical ventilation
  - Re-intubation
- Intensity of perioperative treatment
  - TPOD30
  - Vasopressor dose
  - Inotrope dose
- Fluid status
  - Intravenous fluid volumes infused at 24/48/72 hrs
  - Number of fluid boluses given at 24/48/72 hrs
  - Net fluid balance at 24/48/72 hrs
  - Peak lactate at 24/48/72 hrs
  - Frusemide given (dose)
  - Urine outputs
- Blood products
  - Albumin
  - Packed red blood cells (including pre-transfusion haemoglobin level)
  - Platelets
  - Fresh frozen plasma
  - Cryoprecipitate
- Renal
  - Urine outputs
  - Need for renal replacement therapy
  - Peak creatinine levels
- Cardiovascular monitoring
  - Use of TTE, TOE, PiCCO lines
  - PAC inserted to patient in control group
- Infection

### 16.2.8 Day 30

- Vital status
- DAH30
- MAKE-30
- MACE-30
- Readmission

### 16.2.9 Day 90

- Vital status
- DAH90
- Readmission
- EuroQoL-5d-5l

### 16.2.10 Feasibility data

- Monthly recruitment rate
- Number of patients screened
- Randomised to screened patient ratio
- Reasons for exclusion
- Compliance with protocol
- Protocol deviations and reasons

### 16.2.11 Safety data

- Adverse events up to 30-days
- Serious adverse device events up to 30-days
- Suspected unexpected serious adverse device events up to 30-days

## 16.3 Ethical considerations relating to specific types of data to be collected

Sensitive information: patients will be asked their ethnicity/race. This information is useful in describing the demographics of the included population and is routinely reported in high-quality clinical trials to enable the audience to contextualise the trial and assess external generalisability. To avoid any issues of perceived rudeness, patients will be informed that the sole purpose of collecting this data is to provide the researchers with the ability to describe the demographics of the participants (along with age, sex, etc.).

Health information: we will collect and use a wide range of health information (see CRF) as part of this research. This may also involve us faxing requests for blood test results and discharge summaries from other hospitals. Patients will be informed of this possibility and given the opportunity to ask questions and decline any or all parts of our follow-up procedures.

Data management will be co-ordinated by the coordinating centre including programming (online study database design) and data management support (including data monitoring, database questions, technical issues, data queries, query resolution).

REDCap (Research Electronic Data Capture) is a secure web application used exclusively to support data capture for research studies. REDCap provides: 1) an intuitive interface for validated data entry (with data validation); 2) 256-bit encryption between the data entry client and the server; 3) audit trails for tracking data manipulation and export procedures; 4) automated export procedures for data downloads to common statistical packages (SPSS, SAS, Stata, R et.); and 5) procedures for importing data from external sources; and 6) advanced features such as branching logic, calculated fields and data quality checks.

REDCap is developed and maintained by a team at the Vanderbilt University and licensed free of charge by Melbourne Health. The application and data are housed on servers provided by Melbourne Health. The database is securely backed up off site as per standard hospital procedures for data security. Local support is provided by Melbourne Health.

## 16.4 Source Data Identification Log

The Source Data Identification Log (SDIL) was developed in accordance with RMH Standard Operating Procedure SOP007 and is available upon request.

## 16.5 Data Monitoring Plan

Several procedures to ensure data quality and protocol standardisation will help to minimise bias and to optimise data quality. These include:

- A site initiation teleconference will be conducted before site activation to ensure consistency in procedures
- A detailed data dictionary will define the data to be collected and entered into the eCRF
- The coordinating centre will perform timely validation of data entered into the eCRF, queries and corrections

The study will be monitored by quality control reviews of protocol compliance, data queries, safety-reporting and protocol deviations. On-site monitoring/ source verification will occur for five of the first 20 patients enrolled per centre. These patients will be chosen at random. Further on-site monitoring will be performed thereafter on a case by case basis if quality control issues are flagged by electronic review of data. Medical records, any other relevant source documents and the site investigator files must be made available to the monitoring representative for these monitoring visits during the study and at the completion of the study as needed.

## 16.6 Data Sharing **and** Access Plan

Due to the pilot nature of this study, access to any study data from third parties for secondary research will not be allowed.

# 17 SAFETY PROCEDURES

## 17.1 Background

The PUMA Pilot will employ risk-based safety procedures in accordance with the National Health and Medical Research Council Risk-based Management and Monitoring of Clinical Trials Involving Therapeutic Goods (2018) document). The study Risk Assessment outlines why the PUMA Pilot fits Risk Category Type A (risk comparable to standard medical care) because:

- Both pulmonary artery catheters and central venous access devices are approved in cardiac surgery (and have been for many years), and
- Both devices are considered part of standard medical care of cardiac surgery patients

Accordingly, a lower intensity of safety monitoring is required. Our Safety Committee will perform central monitoring of key data, protocol adherence, and data quality. Site visits will occur quarterly or more frequently if triggered by safety concerns.

It is important to note that nearly all patients meeting study eligibility criteria are currently managed with a pulmonary artery catheter. All adverse events in the pulmonary artery catheter group would therefore have occurred without participation in this study.

This study will compare two alternative versions of standard care for the study population: pulmonary artery catheters (which are currently used in the majority of cases in Victoria and New South Wales) and central venous access devices (a 'central line'), which is preferred in low-risk cases in Queensland and South Australia, and advocated for over pulmonary artery catheters for low risk cases by international clinical practice guidelines. Both devices have

been in routine use for decades and have good safety profiles.

Approximately 100% of patients at RMH and Monash Health who meet eligibility criteria but do not participate in this study will be managed perioperatively with the use of a pulmonary artery catheter (our usual model of care). It is therefore important to approach the assessment of added risk related to involvement in this study based on risks in the control group (those who are randomised to receive a central line). The available evidence suggests patients managed without a pulmonary artery catheter (i.e. with a central line) have less aggressive perioperative management, shorter stays in the intensive care unit, lower rates of complications including acute kidney injury, atrial fibrillation, and blood transfusions; and lower mortality rates. It is important to acknowledge again that multiple international clinical practice guidelines recommend against the routine use of pulmonary artery catheters. See the Background (Section 6.1) of the Protocol for more details.

Possible risks associated with managing patients without a pulmonary artery catheter:

ICU nurses and doctors are used to managing patients with a pulmonary artery catheter. They may feel less confident managing patients with just a central line, particularly in the event of deteriorating patients. It is possible that unfamiliarity in managing deteriorating patients without a pulmonary artery catheter could lead to harm, however there are several mitigating factors (detailed below).

How these risks are minimised:

1. The rationale and design of the PUMA Pilot was presented to the Medical and Nursing director of RMH ICU as well as the Cardiac Surgery Departments of both hospitals where it received unanimous support. Both the Director of ICU and the Nurse Unit Manager at RMH have endorsed the PUMA Pilot. The nursing team leadership are proactively educating and training staff on perioperative management of low risk cases without a pulmonary artery catheter. Below is an excerpt from an unsolicited email from a senior ICU nurse educator who found out about our study:

**'Dear Luke,**

**Just a quick email to say congratulations on the PUMA Pilot and your grant. I just wanted to reach out and say this is one of the most important studies I have seen in a while thank you for trying to answer the question we all ask.**

**Also please let us know if there is anything we can do from a nurse education perspective to assist with your project in ICU.**

**Regards'**

It is also noteworthy that while this is a theoretical risk, that the evidence and clinical practice guidelines all support that these patients should be managed without a pulmonary artery catheter.

2. All patients with a central line will have the central line inserted through a large MAC sheath (as is routine in cardiac surgery) (see Section 11 of the Protocol). It is within the scope of practice of cardiac anaesthetists and intensive care specialists to 'float' a pulmonary artery catheter through a MAC sheath with relative ease and rapidity. Therefore, in the event of patient deterioration where a patient does not have a pulmonary artery catheter available and where treating staff would feel more comfortable managing their patient with a pulmonary artery catheter, one can be inserted. Additionally, noninvasive measurements of cardiac output are allowed in our protocol, so use of a transthoracic echocardiogram or a PiCCO line can also be used in

deteriorating patients.

Risks of inconvenience Cardiac surgery is a highly researched field and patients may be invited to participate in multiple studies. The PUMA Pilot allows co-enrolment where there are no added safety issues and where the patient is not overly burdened by participation.

How this risk was minimised: Our selection of a pragmatic, 'hands-off' design dictates that aside from being randomised to an initial strategy of pulmonary artery catheterisation vs insertion of a central line, no added treatments, investigations, or inpatient contact is required of patients. Patients will be reviewed by telephone at 30 and 90 days after their operation at a time that is convenient for them. We have selected the quality of life questionnaire based on its validity and its brevity; it takes only 2-3 minutes to complete.

## 17.2 Definitions

## The following definitions are provided in the National Health and Medical Research Council’s ‘Safety monitoring and reporting in clinical trials involving therapeutic goods’ document and ISO 14155:2020 ‘Clinical investigation of medical devices for human subjects — Good clinical practice’.^63, 64^

Adverse Event (AE): any untoward medical occurrence in a patient or clinical trial participant administered a medicinal product that does not necessarily have a causal relationship with this treatment.

It is recognised that the patient population with critical illness will experience a number of common aberrations in laboratory values, signs and symptoms due to the severity of the underlying illness and the impact of standard therapies. These will not necessarily constitute an adverse event unless they are considered to be of concern or related to the study or the intervention in the investigator's clinical judgement. In all cases, the condition or disease underlying the symptom, sign or laboratory value should be reported, e.g. renal failure rather than hyperkalaemia, and agitation rather than self-extubation.

Investigational Medical Device (IMD): any instrument, apparatus, implement, machine, appliance, implant, software, material or other similar or related article under study:

a. Intended, by the person under whose name it is or is to be supplied, to be used for human beings for the purpose of one or more of the following:
   1 diagnosis, prevention, monitoring, treatment or alleviation of disease
   2 diagnosis, monitoring, treatment, alleviation of or compensation for an injury or handicap
   3 investigation, replacement or modification of the anatomy or of a physiological process
   4 supporting or sustaining life
   5 control of conception
   6 disinfection of medical devices, and

b. That does not achieve its primary intended action in or on the human body by pharmacological, immunological or metabolic means, but that may be assisted in its intended function by such means.

Adverse Device Effect (ADE): Adverse event related to the use of an investigational medical device.

Device Deficiencies: Inadequacy of a medical device with respect to its identity, quality, durability, reliability, safety or performance.

Serious adverse event (SAE): Any adverse event/adverse reaction that results in death, is life-threatening, requires hospitalisation or prolongation of existing hospitalisation, results in persistent or significant disability or incapacity, or is a congenital anomaly or birth defect

Standard care of patients after cardiac surgery includes a host of complications that fit the definition of an SAE. Death will not be considered an SAE, however will be collected as an outcome as part of the study. Medical and scientific judgement will be exercised by the site chief investigators in deciding whether an adverse event/ reaction will be classified as serious in other situations to avoid over-reporting.

Serious Adverse Device Effect (SADE): An adverse device effect that has resulted in any of the consequences characteristic of a serious adverse event.

Unanticipated Serious Adverse Device Effect (USADE): Serious adverse device effect which by its nature, incidence, severity or outcome has not been identified in the current version of the risk analysis report and is both serious and unexpected.

Suspected unexpected serious adverse reaction (SUSAR): An adverse event that is both serious and unexpected.

Significant safety issue (SSI): A safety issue that could adversely affect the safety of participants or materially impact on the continued ethical acceptability or conduct of the trial.

Urgent safety measure (USM): A measure required to be taken in order to eliminate an immediate hazard to a participant’s health or safety. Note: This type of significant safety issue can be instigated by either the investigator or sponsor and can be implemented before seeking approval from HRECs or institutions

## 17.3 List of potential adverse events

**Complications attributable to both central venous and pulmonary artery catheters:**

- Insertion site haematoma requiring treatment
- Inadvertent arterial puncture
- Internal jugular vein thrombus
- Perforation of the superior vena cava
- Haemothorax or pneumothorax not related to the surgical procedure (i.e. detected prior to sternotomy or postoperatively where the pleura were not breached intraoperatively)
- Central-line associated bloodstream infection (CLABSI)

**Complications attributable to pulmonary artery catheters alone:**

- Ventricular arrhythmia
- Mural thrombus in the right heart
- Air embolism from ruptured balloon
- Pulmonary infarction
- Right bundle branch or complete heart block
- Pulmonary artery perforation, pseudoaneurysm, or rupture
- Tricuspid or pulmonary valve injury
- Knotting
- Right ventricle perforation

NB. All complications must be temporally related to device insertion (i.e. detected within the first 30 days after the index surgical procedure)

## 17.4 Identifying adverse events

Treating doctors will be provided with information on how to report ADEs and SADEs to the CIs after randomisation, and these details will be recorded in the patient’s medical record. Trial staff are responsible for ascertaining whether any ADEs or SADEs have occurred and document these in the CRFs. Patients also have the opportunity to report any ADEs an SADEs to trial staff.

## 17.5 Recording and reporting adverse events

Adverse events/reactions and serious adverse events/reactions will be recorded on a separate case report form. USADES and/or SUSADES should be reported to the coordinating centre within 24 hours of study staff becoming aware of the event.

Minimum information on the report form will include:

- Patient initials and study number
- Nature of the event
- Commencement and cessation of the event
- Outcome of the event
- An investigator’s opinion of the relationship between study involvement and the event (unrelated, possibly, probably or definitively related).
- Whether treatment was required for the event and what treatment was administered.

The coordinating centre staff will be responsible for following-up all USADEs/SUSARs to ensure all details are available. The coordinating centre is also responsible for alerting other participating sites of the report of a SUSAR and informing the TGA within regulatory time frames as set out in Guidance: Safety monitoring and reporting in clinical trials involving therapeutic goods.^65^ It is the responsibility of each principal investigator to inform the local or lead HREC of all SUSAR events which occur at their hospital, in accordance with local requirements. Copies of any reporting and correspondence to and from the local HREC or RGO should also be sent to the coordinating centre.

The sponsors will provide the principal HREC with an annual safety report. Instead of submitting individual reports of AEs, SAEs and SUSARs, the outcomes of the coordinating centre’s analyses of accumulating safety data will be provided to the HREC and the participating institutions.

## 17.6 Assessment of adverse events – Flow Chart

### 17.6.1 Assessment of seriousness

The Investigator will assess the seriousness adverse events as outlined above in Section 15.1.

### 17.6.2 Assessment of causality

The Chief Investigators will assess the likelihood that the AE is related to the IMD using their clinical judgement.

### 17.6.3 Assessment of expectedness

The Chief Investigators will assess the expectedness of the AE based on safety literature for the IMD.

## 17.7 Regulatory reporting requirements

Investigators must adhere to all regulatory reporting requirements relating to the sponsor, instiutions, HREC, and TGA as outlined below.

# 18 STATISTICS AND DATA ANALYSIS

## 18.1 SAMPLE SIZE CALCULATION

The PUMA Pilot requires a total of 150 randomised participants to provide adequate precision around estimates of the feasibility outcomes. Binomial exact (Clopper-Pearson) 95% Confidence Intervals (CIs) for a single proportion will be used to obtain confidence intervals for each feasibility outcome. If the observed proportion is equal to our prior estimate, then the 95% CI will exclude values below the feasibility threshold with 80% power.

| Feasibility outcome | Prior estimate | Feasibility threshold | 95% CI if prior estimate is observed |
| --- | --- | --- | --- |
| Protocol compliance | 0.90 | 0.80 | 0.83 – 0.95 |
| Complete case report form rate | 0.99 | 0.95 | 0.94 – 0.99 |
| Recruitment proportion | 0.60 | 0.30 | 0.51 – 0.69 |
| Acceptability | 0.85 | 0.70 | 0.77 – 0.91 |

**Table 1:** expected precision for feasibility outcomes if the observed proportion is equal to the prior estimate for the overall cohort (150 patients).

## 18.2 STATISTICAL ANALYSIS PLAN

The exact (Clopper-Pearson) two-sided 95% CI for a single proportion will be used to obtain to confidence intervals for each feasibility endpoint. As PUMA Pilot is a feasibility study, our aim is not to conduct hypothesis-testing statistical analyses on clinical outcomes. We will capture and record summary data for all tertiary (clinical) outcomes for the purpose of informing the design and sample size calculation of PUMA II. Analyses will be descriptive, and testing will be reported with caution.

Baseline characteristics of each group were summarised using frequency and percentages for categorical variables and median and interquartile ranges (IQRs) for continuous variables. Binary clinical outcomes were analyzed using generalised linear models with a Poisson distribution with a log link and robust standard errors to estimate risk ratios (RR) and 95% CI. Stratification factors (trial site and surgical urgency) were included as model covariates. Two-sided P values were obtained from Wald tests. Continuous secondary outcomes were compared using quantile regression (τ=0.5) and presented as median differences with 95% CIs. No adjustment for multiplicity will be performed.

## 18.3 MISSING DATA

All reasonable efforts will be made to obtain complete data for every enrolled patient. Where data is considered to be missing at random and data for this variable is missing in no more than 30% of participants, we will perform multiple imputation by chained equations with 20 imputations and 20 iterations after consulting with the Trial Statisticians. Where data cannot be assumed to be missing at random, we will conduct analyses on a complete case-based framework.

# 19 ETHICAL CONDUCT

## 19.1 GUIDING PRINCIPLES

This study is to be performed in accordance with the ethical principles of the Declaration of Helsinki (June 1964 and amended 1975, 1983, 1989, 1996, 2000, 2008 and Note of Clarification 2002 and 2004), ICH GCP Notes for Guidance on Good Clinical Practice (CPMP/ICH/135/95) annotated with Therapeutic Goods Administration comments, NHMRC National Statement on Ethical Conduct in Research Involving Humans 2007 (Updated 2018), ICH GCP Notes for Guidance on Good Clinical Practice (CPMP/ICH/135/95) and the Medical Treatment Planning and Decisions Act 2016.

## 19.2 ETHICAL APPROVALS

The PUMA Pilot must receive full ethical and governance approvals from the Human Research Ethics Committees (HREC) of both RMH, MPH, and Monash Health before commencing.

Each participating site will submit this protocol, and any other relevant study documentation to the responsible local or national constituted Human Research and Ethics Committee (or equivalent). Approval of the protocol, plans for obtaining consent, and related documents will be obtained prior to the start of the study at each site. It is the principal investigator’s responsibility to ensure that all conditions for approval of the study are met and that amendments to the protocol or serious adverse events are also reported to the HREC (or equivalent) as required by that committee.

## 19.3 GOOD CLINICAL PRACTICE TRAINING

All trial staff must hold current Good Clinical Practice (GCP) training in accordance with local HREC policies.

# 20 CONSUMER INVOLVEMENT

The NHMRC Statement on Consumer and Community Involvement in Health and Medical Research emphasised the importance of consumer engagement in producing meaningful research that is relevant to the community.^66^ However, a recent meta-epidemiological study identified that less than 2% of Australian perioperative or surgical RCTs documented any form of consumer engagement.^67^

The PUMA Pilot was designed in collaboration with consumers. The Chair of our Consumer Advisory Committee is Ms Davina Taylor, who is a carer for someone who has had to undergo numerous open heart surgeries, who understands the patient journey through cardiac surgery, and who has provided us with invaluable patient perspectives and contributed to the design of the PUMA Pilot. The Consumer group has led to the inclusion of quality of life, discharge destination (e.g. to a nursing home), and patient experience surveys being added to the trial protocol. Our Consumer Engagement Policy was developed in accordance with the NHMRC National Statement on Ethical Conduct in Human Research, Section 3.1.1 (2018) and the UK National Institute for Health Research 'INVOLVE - Make it clear campaign' (2018). Consumer advisors will be reimbursed at the VCCC rate ($50/hr *pro rata*, plus 3 hours of travel expenses as required). The PUMA Pilot is focused on simplifying care, avoiding unnecessary invasive monitoring and treatments, and delivering outcomes that matter most to patients. We plan to leverage our success with the PUMA Pilot to expand our Consumer Engagement in PUMA II, to ensure our study is aligned with patient preferences and values as it provides world first evidence on this important topic aimed at improving cardiovascular health.

# 21 END OF THE TRIAL

The end of the trial is defined by the completion of the last follow-up on the 150^th^ patient. The Steering Committee may end the trial prematurely for clinical or administrative reasons.

At the end of the trial, the Principal Investigator is responsible for reporting that the trial has ended to the HREC and Sponsor within 30 days. The Principal Investigator is also responsible for updating the status of the PUMA Pilot to the appropriate phase in the relevant trial pages on ANZCTR.org.au.

# 22 ACCESS TO DATA

All trial personnel must comply with the relevant local regulations and legislation. The Chief Investigators are responsible for protecting patient confidentiality to the highest standard, as well as preventing unauthorised access to sensitive patient data. Paper records must be stored in locked cabinets in secure areas. Computers used to collate data and submit eCRFs must be password protected and otherwise secure. Published results will not contain any individual patient data, nor contain any identifiable or re-identifiable patient information. Ownership of trial data resides with the Sponsor at each site. In the event that the Chief Investigators wish to use trial data for a *post hoc* analysis or for other research purposes, for example in an individual patient data meta-analysis, this must be approved by the HREC and the Sponsor.

# 23 DISSEMINATION STRATEGY

## 23.1 COMMUNICATION OF RESULTS TO PARTICIPANTS

Results of the PUMA Pilot will be communicated in plain language to participants who indicate their willingness to receive a newsletter documenting the study results at the time of enrolment.

Announcements of study results will also be made publicly available on a dedicated trial website and through social media.

## 23.2 PUBLICATION

On completion of the PUMA Pilot, we will prepare a detailed Clinical Trial Report (CTR) and submit this to the study sponsor and HRECs within 12 months. We plan to publish this trial in a major surgical or anaesthetic journal such as *JAMA Surgery* or *British Journal of Anaesthesia*. The manuscript must be approved by the Chief Investigators and Writing Committee before submission for publication. We also plan to present the results of the PUMA Pilot at one to two national and international academic conferences. To avoid publication bias, in the event that we are unable to publish the results of the PUMA Pilot in a suitable academic journal, we will make the results freely accessible on an Open Access pre-print server such as medRxiv.org or arXiv.org.

## 23.3 AUTHORSHIP PLAN (PRINCIPAL MANUSCRIPT)

All authors must fulfil International Committee of Medical Journal Editors (ICJME) criteria for authorship. All other personnel who contributed to the PUMA Pilot will be wholeheartedly recognised for their contribution in the published list of Collaborators.

# 24 REFERENCES

1.	Global burden of 369 diseases and injuries in 204 countries and territories, 1990-2019: a systematic analysis for the Global Burden of Disease Study 2019. *Lancet*. Oct 17 2020;396(10258):1204-1222. doi:10.1016/s0140-6736(20)30925-9

2.	Mensah GA, Roth GA, Fuster V. The Global Burden of Cardiovascular Diseases and Risk Factors: 2020 and Beyond. *J Am Coll Cardiol*. Nov 19 2019;74(20):2529-2532. doi:10.1016/j.jacc.2019.10.009

3.	Roth GA, Mensah GA, Johnson CO, et al. Global Burden of Cardiovascular Diseases and Risk Factors, 1990-2019: Update From the GBD 2019 Study. *J Am Coll Cardiol*. Dec 22 2020;76(25):2982-3021. doi:10.1016/j.jacc.2020.11.010

4.	Vervoort D, Meuris B, Meyns B, Verbrugghe P. Global cardiac surgery: Access to cardiac surgical care around the world. *J Thorac Cardiovasc Surg*. Mar 2020;159(3):987-996.e6. doi:10.1016/j.jtcvs.2019.04.039

5.	Vervoort D, Swain JD, Pezzella AT, Kpodonu J. Cardiac Surgery in Low- and Middle-Income Countries: A State-of-the-Art Review. *Ann Thorac Surg*. Apr 2021;111(4):1394-1400. doi:10.1016/j.athoracsur.2020.05.181

6.	Zilla P, Yacoub M, Zühlke L, et al. Global Unmet Needs in Cardiac Surgery. *Glob Heart*. Dec 2018;13(4):293-303. doi:10.1016/j.gheart.2018.08.002

7.	Bowdish ME, D'Agostino RS, Thourani VH, et al. STS Adult Cardiac Surgery Database: 2021 Update on Outcomes, Quality, and Research. *Ann Thorac Surg*. Jun 2021;111(6):1770-1780. doi:10.1016/j.athoracsur.2021.03.043

8.	Shardey G, Tran L, Williams-Spence J, et al. The Australian and New Zealand Society of Cardiac and Thoracic Surgeons

Cardiac Surgery Database Program Annual Report 2019 (2nd ed). Monash University, Department of

Epidemiology and Preventive Medicine,; 2021(12):91.

9.	Rotar EP, Beller JP, Smolkin ME, et al. Prediction of Prolonged Intensive Care Unit Length of Stay Following Cardiac Surgery. *Semin Thorac Cardiovasc Surg*. Mar 6 2021;doi:10.1053/j.semtcvs.2021.02.021

10.	Williams MR, Wellner RB, Hartnett EA, et al. Long-term survival and quality of life in cardiac surgical patients with prolonged intensive care unit length of stay. *Ann Thorac Surg*. May 2002;73(5):1472-8. doi:10.1016/s0003-4975(02)03464-1

11.	Yu PJ, Cassiere HA, Fishbein J, Esposito RA, Hartman AR. Outcomes of Patients With Prolonged Intensive Care Unit Length of Stay After Cardiac Surgery. *J Cardiothorac Vasc Anesth*. Dec 2016;30(6):1550-1554. doi:10.1053/j.jvca.2016.03.145

12.	Hassan A, Anderson C, Kypson A, et al. Clinical outcomes in patients with prolonged intensive care unit length of stay after cardiac surgical procedures. *Ann Thorac Surg*. Feb 2012;93(2):565-9. doi:10.1016/j.athoracsur.2011.10.024

13.	Baxter R, Squiers J, Conner W, et al. Enhanced Recovery After Surgery: A Narrative Review of its Application in Cardiac Surgery. *Ann Thorac Surg*. Jun 2020;109(6):1937-1944. doi:10.1016/j.athoracsur.2019.11.008

14.	Forssmann W. Die Sondierung des Rechten Herzens. *Klinische Wochenschrift*. 1929/11/01 1929;8(45):2085-2087. doi:10.1007/BF01875120

15.	Packy LM, Krischel M, Gross D. Werner Forssmann - A Nobel Prize Winner and His Political Attitude before and after 1945. *Urol Int*. 2016;96(4):379-85. doi:10.1159/000444648

16.	Cournand A, Riley RL, Breed ES, et al. MEASUREMENT OF CARDIAC OUTPUT IN MAN USING THE TECHNIQUE OF CATHETERIZATION OF THE RIGHT AURICLE OR VENTRICLE. *J Clin Invest*. Jan 1945;24(1):106-16. doi:10.1172/jci101570

17.	Raju TN. The Nobel chronicles. 1956: Werner Forssmann (1904-79); André Frédéric Cournand (1895-1988); and Dickinson Woodruff Richards, Jr (1895-1973). *Lancet*. May 29 1999;353(9167):1891. doi:10.1016/s0140-6736(05)75106-0

18.	Swan HJ, Ganz W, Forrester J, Marcus H, Diamond G, Chonette D. Catheterization of the heart in man with use of a flow-directed balloon-tipped catheter. *N Engl J Med*. Aug 27 1970;283(9):447-51. doi:10.1056/nejm197008272830902

19.	Connors AF, Jr., Speroff T, Dawson NV, et al. The effectiveness of right heart catheterization in the initial care of critically ill patients. SUPPORT Investigators. *Jama*. Sep 18 1996;276(11):889-97. doi:10.1001/jama.276.11.889

20.	Harvey S, Harrison DA, Singer M, et al. Assessment of the clinical effectiveness of pulmonary artery catheters in management of patients in intensive care (PAC-Man): a randomised controlled trial. *Lancet*. Aug 6-12 2005;366(9484):472-7. doi:10.1016/s0140-6736(05)67061-4

21.	Wheeler AP, Bernard GR, Thompson BT, et al. Pulmonary-artery versus central venous catheter to guide treatment of acute lung injury. *N Engl J Med*. May 25 2006;354(21):2213-24. doi:10.1056/NEJMoa061895

22.	Richard C, Warszawski J, Anguel N, et al. Early use of the pulmonary artery catheter and outcomes in patients with shock and acute respiratory distress syndrome: a randomized controlled trial. *Jama*. Nov 26 2003;290(20):2713-20. doi:10.1001/jama.290.20.2713

23.	Rajaram SS, Desai NK, Kalra A, et al. Pulmonary artery catheters for adult patients in intensive care. *Cochrane Database Syst Rev*. Feb 28 2013;2013(2):Cd003408. doi:10.1002/14651858.CD003408.pub3

24.	Sandham JD, Hull RD, Brant RF, et al. A randomized, controlled trial of the use of pulmonary-artery catheters in high-risk surgical patients. *N Engl J Med*. Jan 2 2003;348(1):5-14. doi:10.1056/NEJMoa021108

25.	Johnson EK, Nelson CP. Values and pitfalls of the use of administrative databases for outcomes assessment. *J Urol*. Jul 2013;190(1):17-8. doi:10.1016/j.juro.2013.04.048

26.	Youssef N, Whitlock RP. The Routine Use of the Pulmonary Artery Catheter Should Be Abandoned. *Can J Cardiol*. Jan 2017;33(1):135-141. doi:10.1016/j.cjca.2016.10.005

27.	Marik PE. Obituary: pulmonary artery catheter 1970 to 2013. *Ann Intensive Care*. Nov 28 2013;3(1):38. doi:10.1186/2110-5820-3-38

28.	Schwann NM, Hillel Z, Hoeft A, et al. Lack of effectiveness of the pulmonary artery catheter in cardiac surgery. *Anesth Analg*. Nov 2011;113(5):994-1002. doi:10.1213/ANE.0b013e31822c94a8

29.	Xu F, Wang Q, Zhang H, Chen S, Ao H. Use of pulmonary artery catheter in coronary artery bypass graft. Costs and long-term outcomes. *PLoS One*. 2015;10(2):e0117610. doi:10.1371/journal.pone.0117610

30.	Resano FG, Kapetanakis EI, Hill PC, Haile E, Corso PJ. Clinical outcomes of low-risk patients undergoing beating-heart surgery with or without pulmonary artery catheterization. *J Cardiothorac Vasc Anesth*. Jun 2006;20(3):300-6. doi:10.1053/j.jvca.2006.01.017

31.	Chiang Y, Hosseinian L, Rhee A, Itagaki S, Cavallaro P, Chikwe J. Questionable benefit of the pulmonary artery catheter after cardiac surgery in high-risk patients. *J Cardiothorac Vasc Anesth*. Feb 2015;29(1):76-81. doi:10.1053/j.jvca.2014.07.017

32.	Brown JA, Aranda-Michel E, Kilic A, et al. The impact of pulmonary artery catheter use in cardiac surgery. *J Thorac Cardiovasc Surg*. Feb 2 2021;doi:10.1016/j.jtcvs.2021.01.086

33.	Ramsey SD, Saint S, Sullivan SD, Dey L, Kelley K, Bowdle A. Clinical and economic effects of pulmonary artery catheterization in nonemergent coronary artery bypass graft surgery. *J Cardiothorac Vasc Anesth*. Apr 2000;14(2):113-8. doi:10.1016/s1053-0770(00)90001-6

34.	Evans DC, Doraiswamy VA, Prosciak MP, et al. Complications associated with pulmonary artery catheters: a comprehensive clinical review. *Scand J Surg*. 2009;98(4):199-208. doi:10.1177/145749690909800402

35.	Ducatman BS, McMichan JC, Edwards WD. Catheter-induced lesions of the right side of the heart. A one-year prospective study of 141 autopsies. *Jama*. Feb 8 1985;253(6):791-5.

36.	Robin E, Costecalde M, Lebuffe G, Vallet B. Clinical relevance of data from the pulmonary artery catheter. *Crit Care*. 2006;10 Suppl 3(Suppl 3):S3. doi:10.1186/cc4830

37.	Parviainen I, Jakob SM, Suistomaa M, Takala J. Practical sources of error in measuring pulmonary artery occlusion pressure: a study in participants of a special intensivist training program of The Scandinavian Society of Anaesthesiology and Intensive Care Medicine (SSAI). *Acta Anaesthesiol Scand*. May 2006;50(5):600-3. doi:10.1111/j.1399-6576.2006.001008.x

38.	Judge O, Ji F, Fleming N, Liu H. Current use of the pulmonary artery catheter in cardiac surgery: a survey study. *J Cardiothorac Vasc Anesth*. Feb 2015;29(1):69-75. doi:10.1053/j.jvca.2014.07.016

39.	Bignami E, Belletti A, Moliterni P, Frati E, Guarnieri M, Tritapepe L. Clinical practice in perioperative monitoring in adult cardiac surgery: is there a standard of care? Results from an national survey. *J Clin Monit Comput*. Jun 2016;30(3):347-65. doi:10.1007/s10877-015-9725-4

40.	Kastrup M, Carl M, Spies C, Sander M, Markewitz A, Schirmer U. Clinical impact of the publication of S3 guidelines for intensive care in cardiac surgery patients in Germany: results from a postal survey. *Acta Anaesthesiol Scand*. Feb 2013;57(2):206-13. doi:10.1111/aas.12009

41.	Menger J, Edlinger-Stanger M, Dworschak M, Steinlechner B. Postoperative management of patients undergoing cardiac surgery in Austria : A national survey on current clinical practice in hemodynamic monitoring and postoperative management. *Wien Klin Wochenschr*. Dec 2018;130(23-24):716-721. doi:10.1007/s00508-018-1403-3

42.	Singh S. Effectiveness of pulmonary artery catheter in cardiac anaesthesia: a developing countries perspective. 10/30 2020;doi:10.15406/jaccoa.2020.12.00455

43.	Medical Benefits Scheme AIoHaW. Medicare Item Reports. Accessed September 8, 2021, <http://medicarestatistics.humanservices.gov.au/statistics/mbs_item.jsp>

44.	Practice guidelines for pulmonary artery catheterization: an updated report by the American Society of Anesthesiologists Task Force on Pulmonary Artery Catheterization. *Anesthesiology*. Oct 2003;99(4):988-1014. doi:10.1097/00000542-200310000-00036

45.	Lawton JS, Tamis-Holland JE, Bangalore S, et al. 2021 ACC/AHA/SCAI Guideline for Coronary Artery Revascularization: Executive Summary: A Report of the American College of Cardiology/American Heart Association Joint Committee on Clinical Practice Guidelines. *Circulation*. Jan 18 2022;145(3):e4-e17. doi:10.1161/cir.0000000000001039

46.	Wahba A, Milojevic M, Boer C, et al. 2019 EACTS/EACTA/EBCP guidelines on cardiopulmonary bypass in adult cardiac surgery. *Eur J Cardiothorac Surg*. Feb 1 2020;57(2):210-251. doi:10.1093/ejcts/ezz267

47.	Lenkin AI, Kirov MY, Kuzkov VV, et al. Comparison of goal-directed hemodynamic optimization using pulmonary artery catheter and transpulmonary thermodilution in combined valve repair: a randomized clinical trial. *Crit Care Res Pract*. 2012;2012:821218. doi:10.1155/2012/821218

48.	Lee S, Lee SH, Chang BC, Shim JK. Efficacy of Goal-Directed Therapy Using Bioreactance Cardiac Output Monitoring after Valvular Heart Surgery. *Yonsei Med J*. Jul 2015;56(4):913-20. doi:10.3349/ymj.2015.56.4.913

49.	Pearson KS, Gomez MN, Moyers JR, Carter JG, Tinker JH. A cost/benefit analysis of randomized invasive monitoring for patients undergoing cardiac surgery. *Anesth Analg*. Sep 1989;69(3):336-41.

50.	Wisely C. American Society of Anesthesiologists Pulmonary ARtery Catheters for Cardiac Surgery. ABIM Foundation; 2013.

51.	Robin ED. Death by pulmonary artery flow-directed catheter. Time for a moratorium? *Chest*. Oct 1987;92(4):727-31. doi:10.1378/chest.92.4.727

52.	Pulido JN. Commentary: To Swan or not to Swan in cardiac surgery? Narrowing the window of benefit. *J Thorac Cardiovasc Surg*. Feb 19 2021;doi:10.1016/j.jtcvs.2021.02.045

53.	Cowie BS. Does the pulmonary artery catheter still have a role in the perioperative period? *Anaesth Intensive Care*. May 2011;39(3):345-55. doi:10.1177/0310057x1103900305

54.	Thabane L, Ma J, Chu R, et al. A tutorial on pilot studies: the what, why and how. *BMC Med Res Methodol*. Jan 6 2010;10:1. doi:10.1186/1471-2288-10-1

55.	Story DA, Leslie K, French C. Feasibility and pilot studies: small steps before giant leaps. *Anaesth Intensive Care*. Jan 2018;46(1):11-12. doi:10.1177/0310057x1804600103

56.	Nashef SA, Roques F, Sharples LD, et al. EuroSCORE II. *Eur J Cardiothorac Surg*. Apr 2012;41(4):734-44; discussion 744-5. doi:10.1093/ejcts/ezs043

57.	Herdman M, Gudex C, Lloyd A, et al. Development and preliminary testing of the new five-level version of EQ-5D (EQ-5D-5L). *Qual Life Res*. Dec 2011;20(10):1727-36. doi:10.1007/s11136-011-9903-x

58.	Stoppe C, McDonald B, Benstoem C, et al. Evaluation of Persistent Organ Dysfunction Plus Death As a Novel Composite Outcome in Cardiac Surgical Patients. *J Cardiothorac Vasc Anesth*. Jan 2016;30(1):30-8. doi:10.1053/j.jvca.2015.07.035

59.	Vincent JL, Moreno R, Takala J, et al. The SOFA (Sepsis-related Organ Failure Assessment) score to describe organ dysfunction/failure. On behalf of the Working Group on Sepsis-Related Problems of the European Society of Intensive Care Medicine. *Intensive Care Med*. Jul 1996;22(7):707-10. doi:10.1007/bf01709751

60.	Khanna A, English SW, Wang XS, et al. Angiotensin II for the Treatment of Vasodilatory Shock. *N Engl J Med*. Aug 3 2017;377(5):419-430. doi:10.1056/NEJMoa1704154

61.	Khwaja A. KDIGO clinical practice guidelines for acute kidney injury. *Nephron Clin Pract*. 2012;120(4):c179-84. doi:10.1159/000339789

62.	Palevsky PM, Molitoris BA, Okusa MD, et al. Design of clinical trials in acute kidney injury: report from an NIDDK workshop on trial methodology. *Clin J Am Soc Nephrol*. May 2012;7(5):844-50. doi:10.2215/cjn.12791211

63.	Council NHaMR. Guidance: Safety monitoring and reporting in clinical trials involving therapeutic goods. National Health and Medical Research Council; 2016.

64.	(ISO) IOfS. Clinical investigation of medical devices for human subjects — Good clinical practice. 14155:2020. ISO; 2020.

65.	Council NHaMR. Guidance: Safety monitoring and reporting in clinical trials involving therapeutic goods. Canberra: National Health and Medical Research Council; 2016.

66.	Council NHaMR. Statement on consumer and community involvement in health and medical research. Consumers Health Forum of Australia; 2016.

67.	Wallace SKA, Goulding KR, Myles PS. Consumer engagement and patient reported outcomes in perioperative clinical trials in Australia: a systematic review. *ANZ Journal of Surgery*. n/a(n/a)doi:<https://doi.org/10.1111/ans.17897>

# 25 APPENDICES

**Health Questionnaire**

**English version for Australia**

**VERSION FOR INTERVIEWER ADMINISTRATION**

*Note to interviewer: although allowance should be made for the interviewer’s particular style of speaking, the wording of the questionnaire instructions should be followed as closely as possible. In the case of the EQ-5D-5L descriptive system on page 2 of the questionnaire, the precise wording must be followed.*

*If the respondent has difficulty choosing a response, or asks for clarification, the interviewer should repeat the question word for word and ask the respondent to answer in a way that most closely resembles his or her thoughts about his or her health today.*

**INTRODUCTION**

*(Note to interviewer: please read the following to the respondent.)*

**We are trying to find out what you think about your health. I will explain what to do as I go along, but please interrupt me if you do not understand something or if things are not clear to you. There are no right or wrong answers. We are interested only in your personal view.**

**First, I am going to read out some questions. Each question has a choice of five answers. Please tell me which answer best describes your health TODAY.**

**Do not choose more than one answer in each group of questions.**

(Note to interviewer: first read all five options for each question. Then ask the respondent to choose which one applies to him/herself. Repeat the question and options if necessary. Mark the appropriate box under each heading. You may need to remind the respondent regularly that the timeframe is TODAY.)

EQ-5D DESCRIPTIVE SYSTEM

| **First, I would like to ask you about MOBILITY. Would you say that:** | |
| --- | --- |
| 1. You have no problems with walking around? | ❑ |
| 2. You have slight problems with walking around? | ❑ |
| 3. You have moderate problems with walking around? | ❑ |
| 4. You have severe problems with walking around? | ❑ |
| 5. You are unable to walk around? | ❑ |

| **Next, I would like to ask you about PERSONAL CARE. Would you say that:** | |
| --- | --- |
| 1. You have no problems with washing or dressing yourself? | ❑ |
| 2. You have slight problems with washing or dressing yourself? | ❑ |
| 3. You have moderate problems with washing or dressing yourself? | ❑ |
| 4. You have severe problems with washing or dressing yourself? | ❑ |
| 5. You are unable to wash or dress yourself? | ❑ |

| **Next, I would like to ask you about USUAL ACTIVITIES, for example work, study, housework, family or leisure activities. Would you say that:** | |
| --- | --- |
| 1. You have no problems doing your usual activities? | ❑ |
| 2. You have slight problems doing your usual activities? | ❑ |
| 3. You have moderate problems doing your usual activities? | ❑ |
| 4. You have severe problems doing your usual activities? | ❑ |
| 5. You are unable to do your usual activities? | ❑ |

| **Next, I would like to ask you about PAIN OR DISCOMFORT. Would you say that:** | |
| --- | --- |
| 1. You have no pain or discomfort? | ❑ |
| 2. You have slight pain or discomfort? | ❑ |
| 3. You have moderate pain or discomfort? | ❑ |
| 4. You have severe pain or discomfort? | ❑ |
| 5. You have extreme pain or discomfort? | ❑ |

| **Finally, I would like to ask you about ANXIETY OR DEPRESSION. Would you say that:** | |
| --- | --- |
| 1. You are not anxious or depressed? | ❑ |
| 2. You are slightly anxious or depressed? | ❑ |
| 3. You are moderately anxious or depressed? | ❑ |
| 4. You are severely anxious or depressed? | ❑ |
| 5. You are extremely anxious or depressed? | ❑ |

EQ-5D VAS

- Now, I would like to ask you to say how good or bad your health is TODAY.

- **I would like you to picture in your mind a vertical line that is numbered from 0 to 100.**

*(Note to interviewer: if interviewing face-to-face, please show the respondent the VAS line.)*

- 100 at the top of the line means the best health you can imagine.

0 at the bottom of the line means the worst health you can imagine.

- **I would now like you to tell me the point on this line where you would put your health TODAY.**

*(Note to interviewer: mark the line at the point indicating the respondent’s health today. Now, please write the number you marked on the line in the box below.)*

The best health you can imagine

THE RESPONDENT’S HEALTH TODAY =

**Thank you for taking the time to answer these questions.**

**Product information**

The worst health you can imagine

1. This protocol has been reported in accordance with the SPIRIT 2013 reporting guidelines. Chan et al. SPIRIT 2013 statement: defining standard protocol items for clinical trials. *Ann Intern Med* 2013;158(3):200-7).

<https://www.youtube.com/" \o "YouTube Home>
