# Supplementary material for: Pulmonary artery catheters or central venous catheters for cardiac surgery: the PUMA Pilot randomised clinical trial
Source: Anaesthesia. 2026 Jun 28;81(10):1358–66. doi: 10.1111/anae.70282 (PMC13551237; doi:10.1111/anae.70282)
Supplement: Supplementary file 2 [file ANAE-81-1358-s002.docx]

**Table S1:** Expected precision for feasibility outcomes if the observed proportion is equal to the prior estimate for the overall cohort. Values are proportion and 95% CI

| **Feasibility outcome** | **Prior estimate** | **Feasibility threshold** | **95% CI if prior estimate is observed** |
| --- | --- | --- | --- |
| Protocol compliance | 0.90 | 0.80 | 0.83 – 0.95 |
| Complete case report form rate | 0.99 | 0.95 | 0.94 – 0.99 |
| Recruitment proportion | 0.60 | 0.30 | 0.51 – 0.69 |
| Acceptability | 0.85 | 0.70 | 0.77 – 0.91 |

# Table S2: Patient Baseline Characteristics

| **Characteristic** | **Pulmonary artery catheter group**  **(N=76)** | **Central venous catheter group**  **(N=73)** |
| --- | --- | --- |
| **Age, y** | 66 (57–70 [24-80]) | 64 (58–72 [35-83]) |
| **Female sex** | 8 (10.5) | 10 (13.7) |
| **BMI** | 28 (26–32 [19-44]) | 28 (25–32 [20-61]) |
| **Ethnicity**  White  South Asian  East Asian  Indigenous  Black  Other | 50 (65.8)  13 (17.1)  4 (5.3)  0 (0)  1 (1.3)  8 (10.5) | 56 (76.7)  5 (6.8)  1 (1.4)  2 (2.8)  1 (1.4)  8 (10.9) |
| **Trial site**  Victorian Heart Hospital  Royal Melbourne Hospital  Jessie McPherson Private Hospital | 59 (77.6)  14 (18.4)  3 (3.9) | 57 (78.1)  13 (17.8)  3 (4.1) |
| **Surgery type**  CABG  AVR  Thoracic aorta | 70 (92.1)  6 (7.9)  0 (0) | 68 (93.2)  5 (6.8)  0 (0) |
| **Urgency**  Elective  Urgent | 39 (51.3)  37 (48.7) | 40 (54.8)  33 (45.2) |
| **EuroSCORE II** | 0.94 (0.69–1.26 [0.50-1.98]) | 0.97 (0.79–1.29 [0.50-1.89]) |
| **Cardiopulmonary bypass used** | 73 (96.1) | 70 (95.9) |
| **Cardiopulmonary bypass time, mins** | 95.1 (25.6) | 102 (34.8) |
| **Comorbidities**  AMI within 90d  Atrial fibrillation  Chronic kidney disease  COPD  Current smoker  Diabetes  Dyslipidemia  Heart failure  Hypertension  Extracardiac arteriopathy  Stroke | 27 (35.5)  2 (2.6)  8 (10.5)  5 (6.6)  11 (14.5)  25 (32.9)  63 (82.9)  1 (1.3)  59 (77.6)  3 (3.9)  2 (2.6) | 26 (35.6)  2 (2.7)  14 (19.2)  5 (6.8)  10 (13.7)  19 (26.0)  58 (79.5)  3 (4.1)  53 (72.6)  5 (6.8)  8 (11.0) |
| **Preoperative LVEF**  >50%  31–50%  <30% | 68 (89.5)  8 (10.5)  0 (0) | 63 (86.3)  10 (13.7)  0 (0) |
| **Preoperative medication**  ACEi or ARNi  Aspirin  Beta blocker  Clopidogrel  Diuretic  Insulin  Oral hypoglycemic agent  Statin | 45 (59.2)  71 (93.4)  48 (63.2)  8 (10.5)  9 (11.8)  8 (10.5)  23 (30.3)  68 (89.5) | 44 (60.3)  69 (94.5)  42 (57.5)  15 (20.5)  5 (6.8)  8 (11.0)  17 (23.3)  69 (94.5) |
| **Preoperative labs**  Haemoglobin (g/L)  Platelets (×10^-9^/L)  eGFR (L/min/1.73m^2^) | 143.1 (13.3)  234.4 (63.0)  77.6 (12.9) | 138.1 (13.4)  236.9 (68.5)  78.0 (14.3) |

**Abbreviations:** ACEi; angiotensin converting enzyme inhibitor; AMI, acute myocardial infarction; ARNi, angiotensin-neprilysin inhibitor; AVR, aortic valve replacement; BMI, body mass index; CABG, coronary artery bypass graft surgery; COPD, chronic obstructive pulmonary disease; eGFR, estimated glomerular filtration rate; LVEF, left ventricular ejection fraction Values are number (proportion), median (IQR [range]) and mean (SD)

**Table S3: Procedural events.**

| **Outcome** | **No. (%)** | | **RR (95% CI)** | **P value** |
| --- | --- | --- | --- | --- |
|  | **PAC Group**  (n = 76) | **CVC Group**  (n = 73) |  |  |
| Return to theatre | 1/76 (1.3%) | 0/73 (0.0%) | - | - |
| Re-intubation | 0/76 (0.0%) | 2/73 (2/73.7%) | - | - |
| Postoperative use of echocardiography | 11/76 (14.5%) | 16/73 (21.9%) | 0.66 (0.33–1.30) | 0.29 |
| Use of non-invasive cardiac output monitoring device | 0/76 (0.0%) | 0/73 (0.0%) | - | - |
| ICU readmission | 3/76 (3.9%) | 4/73 (5.5%) | 0.70 (0.16–2.97) | 0.63 |

Abbreviations: CI, confidence interval; CVC, central venous catheter; ICU, intensive care unit; PAC, pulmonary artery catheter; RR, relative risk.

Values are number (proportion), and RR (95% CI)

Adjusted risk ratios were not estimable for binary outcomes where one or both groups had zero events.

**Table S4: Major complications**

| **Outcome** | **No. (%)** | | **RR (95% CI)** | **P value** |
| --- | --- | --- | --- | --- |
|  | **PAC Group**  (n = 76) | **CVC Group**  (n = 73) |  |  |
| Acute kidney injury | 26/76 (34.2%) | 14/73 (19.2%) | 1.80 (1.03–3.14) | 0.04 |
| New renal replacement therapy to 30 d | 0/76 (0.0%) | 0/73 (0.0%) | - | - |
| Serious infection | 5/76 (6.6%) | 7/73 (9.6%) | 0.68 (0.23–2.03) | 0.49 |
| Post-operative atrial fibrillation | 28/76 (36.8%) | 24/73 (32.9%) | 1.10 (0.71–1.70) | 0.66 |
| Major adverse kidney events at 30 d | 1/75 (1.3%) | 0/73 (0.0%) | - | - |
| Major adverse cardiac events at 30 d | 0/75 (0.0%) | 0/73 (0.0%) | - | - |
| All-cause mortality in hospital | 0/76 (0.0%) | 0/73 (0.0%) | - | - |
| All-cause mortality at 30 d | 0/75 (0.0%) | 0/73 (0.0%) | - | - |
| All-cause mortality at 90 d | 0/75 (0.0%) | 0/72 (0.0%) | - | - |

**.** Abbreviations: CI, confidence interval; CVC, central venous catheter; PAC, pulmonary artery catheter; RR, risk ratio.

Values are number (proportion), and RR (95% CI)

**Table S5: Allogeneic blood products and albumin.** Abbreviations: CI, confidence interval; CVC, central venous catheter; PAC, pulmonary artery catheter; RR, risk ratio.

Values are number (proportion), and RR (95% CI)

Due to the low number of patients receiving these interventions, results are presented as the proportion of patients receiving at least 1 unit of the intervention, rather than as continuous outcomes.

| **Outcome** | **No. (%)** | | **RR (95% CI)** | **P value** |
| --- | --- | --- | --- | --- |
|  | **PAC Group**  (n = 76) | **CVC Group**  (n = 73) |  |  |
| Packed red blood cells | 10/76 (13.2%) | 13/73 (17.8%) | 0.73 (0.35–1.56) | 0.42 |
| Fresh frozen plasma | 5/76 (6.6%) | 3/73 (4.1%) | 1.60 (0.39–6.55) | 0.51 |
| Platelets | 5/76 (6.6%) | 3/73 (4.1%) | 1.60 (0.40–6.38) | 0.50 |
| Cryoprecipitate | 5/76 (6.6%) | 3/73 (4.1%) | 1.59 (0.38–6.58) | 0.52 |
| Prothrombin complex concentrate | 0/76 (0.0%) | 1/73 (1.4%) | - | - |
| Albumin | 8/76 (10.5%) | 9/73 (12.3%) | 0.85 (0.35–2.09) | 0.80 |

**Table S6: Diuretics, fluid balance and lactate.** Abbreviations: CI, confidence interval; CVC, central venous catheter; IQR, interquartile range; MD, median difference; PAC, pulmonary artery catheter. Values are median (IQR [range])

| **Outcome** | **Median (IQR [range])** | | **MD (95% CI)** | **P value** |
| --- | --- | --- | --- | --- |
|  | **PAC Group**  (n = 76) | **CVC Group**  (n = 73) |  |  |
| Cumulative frusemide to 7 d, mg | 280.00 (200.00–400.00 [0.00-1040.00]) | 280.00 (200.00–400.00 [0.00-1469.40]) | 0.00 (-45.18–45.18) | 1.00 |
| Net fluid balance at 24 h, mL | -1185.00 (-1906.42 to -418.37 [-7270.00-3958.00]) | -1423.22 (-1805.00 to -653.87 [-3430.98–1517.89] | 238.22 (-92.85–569.29) | 0.16 |
| Net fluid balance at 48 h, mL | -1085.00 (-1413.50 to -759.34 [-3680.00–566.00]) | -1015.00 (-1491.29 to -776.33 [-3907.00–1088.96]) | -70.00 (-297.74–157.74) | 0.55 |
| Net fluid balance at 72 h, mL | -688.03 (-1491.70 to -180.00 [-5036.00–332.00]) | -1350.00 (-2145.00 to -700.00 [-4374.00–166.00]) | 641.00 (187.75–1094.25) | 0.006 |
| Peak lactate at 24 h, mmol/L | 1.90 (1.40–2.58 [0.90–7.50]) | 2.00 (1.50–3.00 [0.60–32.00]) | -0.10 (-0.46–0.26) | 0.58 |
| Peak lactate at 48 h, mmol/L | 1.30 (1.10–1.78  [0.70–4.50]) | 1.60 (1.20–2.40 [0.70–4.70]) | -0.30 (-0.62–0.02) | 0.07 |
| Peak lactate at 72 h, mmol/L | 1.20 (0.97–1.63 [0.60–3.10]) | 1.30 (1.00–1.60 [0.80–2.70]) | -0.10 (-0.37–0.17) | 0.468 |

**Table S7: Vasopressor and inotrope requirements.** Abbreviations: CI, confidence interval; CVC, central venous catheter; IQR, interquartile range; MD, median difference; PAC, pulmonary artery catheter; RR, risk ratio.

Values are number (proportion), and RR (95% CI)

Due to the low number of patients receiving these interventions, results for all vasoactive drugs except norepinephrine are presented as the proportion of patients receiving any of the intervention, rather than as continuous outcomes.

| **Outcome** | **No. (%)** | | **RR (95% CI)** | **P value** |
| --- | --- | --- | --- | --- |
|  | **PAC Group**  (n = 76) | **CVC Group**  (n = 73) |  |  |
| Norepinephrine | 67/76 (88.2%) | 58/73 (79.5%) | 1.11 (0.96–1.28) | 0.15 |
| Epinephrine | 10/76 (13.2%) | 6/73 (8.2%) | 1.58 (0.61–4.11) | 0.35 |
| Vasopressin | 1/76 (1.3%) | 1/73 (1.4%) | 1.00 (0.07–15.21) | 1.00 |
| Dopamine | 0/76 (0.0%) | 0/73 (0.0%) | - | - |
| Dobutamine | 2/76 (2.6%) | 5/73 (6.8%) | 0.39 (0.08–1.91) | 0.25 |
| Milrinone | 1/76 (1.3%) | 0/73 (0.0%) | - | - |
| Levosimendan | 0/76 (0.0%) | 1/73 (1.4%) | - | - |
|  | **Median (IQR [range])** | | **MD (95% CI)** | **P value** |
| Cumulative norepinephrine dose at 7 d, µg | 1386.00 (296.40–3440.10 [0.00–47071.10]) | 2476.20 (258.60–4980.00 [0.00–31941.00]) | -1084.20 (-2109.36–59.04) | 0.04 |

**Table S8: Course of recovery.** Abbreviations: CI, confidence interval; CVC, central venous catheter; ICU, intensive care unit; MD, median difference; PAC, pulmonary artery catheter; RR, risk ratio; SOFA, Sequential Organ Failure Assessment score; TPOD_30,_ Time in perioperative organ dysfunction at 30 days. Values are number (proportion), median (IQR [range]) and RR (95% CI)

| **Outcome** | **No. (%)** | | **RR (95% CI)** | **P value** |
| --- | --- | --- | --- | --- |
|  | **PAC Group**  (n = 76) | **CVC Group**  (n = 73) |  |  |
| **Discharge disposition** | | | | |
| Discharged home | 74/76 (97.4%) | 68/73 (93.2%) | 1.05 (0.98–1.12) | 0.18 |
| Discharged to rehabilitation facility | 2/76 (2.6%) | 3/73 (4.1%) | 0.73 (0.20-2.69) | 0.63 |
| Discharged to other hospital | 0/76 (0.0%) | 1/73 (1.4%) | - | - |
| Discharged to supported accommodation | 0/76 (0.0%) | 1/73 (1.4%) | - | - |
| Discharged to nursing facility | 0/76 (0.0%) | 0/73 (0.0%) | - | - |
| Discharged to other destination | 0/76 (0.0%) | 0/73 (0.0%) | - | - |
|  | **Median Score (IQR [range])** | | **MD (95% CI)** | **P value** |
| **SOFA Scores** | | | | |
| SOFA score on day 1 | 8.00 (6.00–9.75 [3.00–15.00]) | 8.00 (7.00–11.00 [3.00–14.00]) | 0.00 (-2.23–2.23) | 1.00 |
| SOFA score on day 2 | 4.00 (4.00–6.50 [3.00–10.00]) | 7.50 (4.00–10.25 [3.00–13.00]) | -3.00 (-6.92–0.92) | 0.13 |
| SOFA on day 3 | 4.00 (4.00–10.00 [4.00–16.00]) | 5.00 (4.00–8.50 [4.00–12.00]) | -1.00 (-4.57–2.57) | 0.58 |
| SOFA on day 4 | 4.00 (4.00–6.00 [4.00–10.00]) | 4.50 (4.00–7.00 [4.00–11.00]) | 0.00 (-3.01–3.01) | 1.00 |
| SOFA on day 5 | 6.00 (4.00–8.00 [4.00–17.00]) | 5.00 (4.25–5.00 [4.00–11.00]) | 1.00 (-3.42–5.42) | 0.66 |
| SOFA on day 6 | 7.00 (6.50–12.00 [6.00–17.00]) | 4.50 (4.00–6.50 [4.00–10.00]) | 2.00 (-6.13–10.13) | 0.63 |
| **Other recovery processes** | | | | |
| TPOD_30_, hours | 16.94 (9.56–26.00 [2.47–160.00]) | 15.60 (9.38–26.72 [2.43–200.00]) | 1.27 (-2.50–5.04) | 0.51 |
| ICU length of stay, hours | 25.00 (22.00–30.00  [13.00–236.00]) | 28.00 (22.00–50.00 [16.00–189.00]) | -3.00 (-9.83–3.83) | 0.39 |
| Hospital length of stay, days | 6.29 (5.33 – 7.33 [4.00-17.25]) | 6.65 (6.04 – 8.14 [4.00-21.33]) | -0.13 (-1.11 – 0.86) | 0.80 |
| Duration of any vasoactive or inotrope infusion, hours | 14.48 (6.66–21.95  [0.00–159.50]) | 14.53 (5.57–23.37 [0.00–83.70]) | 0.20 (-4.34–4.74) | 0.93 |
| Duration of mechanical ventilation, hours | 6.00 (5.00–9.50 [2.00–161.00]) | 7.00 (5.00–9.00 [2.00–81.00]) | -1.00 (-2.35–0.35) | 0.15 |

**Table S9: Patient-centred outcomes.** Abbreviations: CI, confidence interval; CVC, central venous catheter; EQ-5D-5L, European Quality of Life 5 Dimensions 5 Levels; IQR, interquartile range; MD, median difference; PAC, pulmonary artery catheter; VAS, Visual Analogue Scale.

Values are median (IQR [range])

**a** Change in the EQ-5D-5L Index was calculated as the EQ-5D-5L index value at 90-day follow-up minus the index value at enrolment. The index is a single value summary of the 5 domain scores comprising the questionnaire. Positive values indicate improvement in health-related quality of life whereas negative values indicate worsening.

**b** Change in the EQ-5D VAS was calculated as the EQ-5D-5L VAS score at 90-day follow up minus the VAS score at enrolment. Positive values denote improvement in self-reported health status.

| **Outcome** | **Median (IQR [range])** | | **MD (95% CI)** | **P value** |
| --- | --- | --- | --- | --- |
|  | **PAC Group**  (n = 76) | **CVC Group**  (n = 73) |  |  |
| Days alive and at home at 30 d, d | 23.70 (21.93–24.68 [6.95–26.00]) | 22.87 (20.84–23.93 [8.65–25.80]) | 0.78 (-0.44–2.01) | 0.21 |
| Days alive and at home to 90 d, d | 83.03 (81.79–84.68 [66.99–86.00]) | 82.61 (80.71–83.84 [54.94–85.80]) | 0.42 (-0.79–1.63) | 0.50 |
| Change in EQ-5D-5L Index Score^a^ at 90 d post-surgery | 0.032 (-0.001–0.076 [-0.837-0.844]) | 0.032 (0.000–0.103 [-0.302-0.549]) | -0.000 (-0.025 to 0.025) | 1.00 |
| Change in EQ-5D-5L VAS score at 90 d post-surgery^b^ | 10.00 (0.00–25.00 [-25.00–50.00]) | 8.00 (0.00–20.00 [-30.00–58.00]) | 2.00 (-3.98–7.98) | 0.51 |
