# Supplementary material for: Pulmonary artery catheters or central venous catheters for cardiac surgery: the PUMA Pilot randomised clinical trial
Source: Anaesthesia. 2026 Jun 28;81(10):1358–66. doi: 10.1111/anae.70282 (PMC13551237; doi:10.1111/anae.70282)
Supplement: Supplementary file 3 [file ANAE-81-1358-s003.docx]

# **Appendix S2**: List of Pilot Clinical and Safety Outcomes

## 2.1 Clinical Outcomes

#### ICU Outcomes

| **Time in postoperative organ dysfunction (T_POD_) at 30 days [1]** | **Definition:** the combined duration of requirement of one or more of the following life-sustaining therapies: mechanical ventilation (not including non-invasive ventilation), vasopressor or inotrope therapy, mechanical circulatory support (e.g. extracorporeal membrane oxygenation or intra-aortic balloon pump), and continuous renal replacement therapy or new intermittent haemodialysis.  **Timing**: from admission to ICU until 30 days. |
| --- | --- |
| **ICU Length of Stay** | **Definition:** the number of hours from the index admission to ICU until the patient is discharged from the ICU |
| **SOFA Score** | **Definition:** the Sequential Organ Failure Assessment (SOFA) score calculated using validated methods [2].  **Timing:** daily from admission to ICU until 7 days. |
| **Duration of vasopressor or inotropic support** | **Definition:** the total number of hours a patient is administered vasopressor and/or inotropic support. Vasopressor support is defined by any use of: norepinephrine, vasopressin, metaraminol, or phenylephrine. Inotrope support is defined by any use of: epinephrine, dobutamine, dopamine, levosimendan, milrinone, or isoprenaline.  **Timing**: from admission to ICU until 7 days. |
| **Cumulative dose of vasoactive drugs** | Definition: Cumulative dose of vasopressor drugs, measured in noradrenaline equivalents using established methods [3], and inotropic drugs.  **Timing**: from admission to ICU until 7 days. |
| **Cumulative dose of diuretic drugs** | **Definition:** cumulative dose (in mg) of frusemide administered orally or intravenously.  **Timing:** from admission to ICU until 7 days. |
| **Cumulative dose of albumin** | **Definition:** cumulative dose (in g) of albumin administered.  **Timing:** from admission to ICU until 7 days. |
| **Net fluid balance at 24, 48, and 72 hours** | **Definition:** the cumulative volume of fluids administered to the patient from any source minus the cumulative measured volume of fluids removed from the patient by any source.  **Timing:** from admission to ICU to 24, 48, and 72 hours. |
| **Peak lactate at 24, 48, and 72 hours** | **Definition:** the highest value recorded of plasma lactate levels in mmol/L.  **Timing:** from admission to ICU to 24, 48, and 72 hours. |
| **Return to theatre** | **Definition:** return to theatre for any cardiothoracic reoperation.  **Timing:** from admission to ICU until 7 days. |
| **Duration of mechanical ventilation** | **Definition:** the number of hours from when the patient is first placed on mechanical ventilation in the operating theatre until final extubation (separate periods of intubation and mechanical ventilation are additive).  **Timing**: during the index ICU admission. |
| **Re-intubation** | **Definition**: re-insertion of an endotracheal tube after one is removed.  **Timing**: during the index ICU admission. |
| **Renal replacement therapy** | **Definition:** the patient receives any form of renal replacement therapy which was not required preoperatively.  **Timing:** from admission to the ICU until 30 days have elapsed. |
| **Utilisation of echocardiography** | **Definition:** the number of times each of the following are performed: bedside transthoracic echocardiography (TTE), formal diagnostic TTE performed by a sonographer or cardiologist, and transesophageal echocardiography.  **Timing:** from admission to ICU until 7 days. |
| **Use of non-invasive cardiac output monitoring devices** | **Definition:** use of any non-invasive cardiac output monitoring device (e.g. pulse contour devices [PiCCO], bioreactance devices, etc.).  **Timing:** from admission to ICU until 7 days. |
| **Readmission to the ICU** | **Definition:** the patient is readmitted to the ICU after they have been discharged and transferred to another setting.  **Timing:** from discharge from ICU until the patient is discharged from hospital. |

#### Hospital Outcomes

| **Blood product transfusion** | **Definition:** the volume in millilitres transfused of each of the following blood products: packed red blood cells, platelets, cryoprecipitate, fresh frozen plasma, and prothrombin complex concentrates.  **Timing**: from start of surgery until hospital discharge |
| --- | --- |
| **Serious infection** | **Definition:** a composite of sepsis, deep sternal wound infection, graft site infection, radiographically and clinically proven pneumonia, and central-line associated blood stream infection (CLABSI)  **Timing:** from admission to the ICU until discharge from hospital. |
| **Post-operative atrial fibrillation** | **Definition:** the development of new-onset and persistent (lasting at least 60 minutes or requiring any intervention) atrial fibrillation from admission to ICU until 7 days have elapsed. |
| **Acute kidney injury** | **Definition**: acute kidney injury, defined by KDIGO Criteria, occurring during the hospital admission [4]  **Timing:** from induction of anaesthesia until discharge from hospital |
| **In-hospital mortality** | **Definition:** death due to any cause occurring during the index hospital admission  **Timing:** from induction of anaesthesia until discharge from hospital |
| **Hospital length of stay** | **Definition:** the number of hours from admission to ICU until the patient is discharged from hospital. |
| **Discharge destination** | **Definition:** the type of place the patient is discharged to. Categories include: rehabilitation facility/step-down care, another hospital, the patient’s home, supported accommodation, nursing facility, or other. |

#### 30-Day Outcomes

| **Days alive and out of hospital at 30 days** | **Definition:** the number of days from admission to ICU until 30 days have elapsed or the patient dies, minus the number of days the patient spent admitted to any hospital during this period (either during the index hospitalization or any subsequent readmission.)  **Timing:** from admission to ICU until 30 days. |
| --- | --- |
| **All-cause mortality** | **Definition:** death due to any cause.  **Timing:** from the start of anaesthetic care until 30 days have elapsed. |
|  |  |
| **Hospital readmission** | **Definition**: the patient is readmitted to any hospital for any reason.  **Timing:** from admission to ICU until 30 days have elapsed. |
| **Major Adverse Cardiac Events (MACE-30)** | **Definition**: a composite of all-cause mortality, stroke, AMI, repeat revascularization procedure, and readmission to hospital due to heart failure  **Timing:** from admission to the intensive care unit until 30 days have elapsed. |
| **Major Adverse Kidney Events (MAKE-30)[5]** | **Definition:** a composite of all-cause mortality, need for renal replacement therapy, and persistent renal dysfunction[5]  **Timing:** from admission to the intensive care unit until 30 days have elapsed. |

#### 90-Day Outcomes

| **All-cause mortality** | **Definition:** death due to any cause.  **Timing:** from the start of anaesthetic care until 90 days have elapsed. |
| --- | --- |
| **Change in quality of life** | **Definition:** EuroQoL – 5 dimension – 5 level (EQ-5D-5L) score at 90-days less the EQ-5D-5L score at enrolment [6].  **Timing:** assessed at enrolment and at 90 days by telephone interview. |
| **Days alive and out of hospital at 90 days** | **Definition:** the number of days from admission to ICU until 90 days have elapsed or the patient dies, minus the number of days the patient spent admitted to any hospital during this period (either during the index hospitalization or any subsequent readmission.)  **Timing:** from admission to ICU until 90 days. |

## 2.2 Safety Outcomes

**Complications attributable to both central venous and pulmonary artery catheters:**

- Insertion site haematoma requiring treatment
- Inadvertent arterial puncture
- Internal jugular vein thrombus
- Perforation of the superior vena cava
- Haemothorax or pneumothorax not related to the surgical procedure (i.e. detected prior to sternotomy or postoperatively where the pleura were not breached intraoperatively)
- Central-line associated bloodstream infection (CLABSI)

**Complications attributable to pulmonary artery catheters alone:**

- Ventricular arrhythmia
- Mural thrombus in the right heart
- Air embolism from ruptured balloon
- Pulmonary infarction
- Right bundle branch or complete heart block
- Pulmonary artery perforation, pseudoaneurysm, or rupture
- Tricuspid or pulmonary valve injury
- Knotting
- Right ventricle perforation

NB. All complications must be temporally related to device insertion (i.e. detected within the first 30 days after the index surgical procedure)

**References**

1.	Stoppe C, McDonald B, Benstoem C, et al. Evaluation of persistent organ dysfunction plus death as a novel composite outcome in cardiac surgical patients. *J Cardiothorac Vasc Anesth* 2016; **30:** 30-8. 10.1053/j.jvca.2015.07.035

2.	Vincent JL, Moreno R, Takala J, et al. The sofa (sepsis-related organ failure assessment) score to describe organ dysfunction/failure. On behalf of the working group on sepsis-related problems of the european society of intensive care medicine. *Intensive Care Med* 1996; **22:** 707-10. 10.1007/bf01709751

3.	Khanna A, English SW, Wang XS, et al. Angiotensin ii for the treatment of vasodilatory shock. *N Engl J Med* 2017; **377:** 419-30. 10.1056/NEJMoa1704154

4.	Khwaja A. Kdigo clinical practice guidelines for acute kidney injury. *Nephron Clin Pract* 2012; **120:** c179-84. 10.1159/000339789

5.	Palevsky PM, Molitoris BA, Okusa MD, et al. Design of clinical trials in acute kidney injury: Report from an niddk workshop on trial methodology. *Clin J Am Soc Nephrol* 2012; **7:** 844-50. 10.2215/cjn.12791211

6.	Herdman M, Gudex C, Lloyd A, et al. Development and preliminary testing of the new five-level version of eq-5d (eq-5d-5l). *Qual Life Res* 2011; **20:** 1727-36. 10.1007/s11136-011-9903-x
